# Assessment of Environmental Pollution and Human Exposure to Pesticides by Wastewater Analysis in a Seven-Year Study in Athens, Greece

**DOI:** 10.3390/toxics9100260

**Published:** 2021-10-11

**Authors:** Nikolaos I. Rousis, Maria Denardou, Nikiforos Alygizakis, Aikaterini Galani, Anna A. Bletsou, Dimitrios E. Damalas, Niki C. Maragou, Kevin V. Thomas, Nikolaos S. Thomaidis

**Affiliations:** 1 Laboratory of Analytical Chemistry, Department of Chemistry, National and Kapodistrian University of Athens, Panepistimiopolis Zografou, 15771 Athens, Greece; mariadnrd@chem.uoa.gr (M.D.); nalygizakis@chem.uoa.gr (N.A.); kate-galani@chem.uoa.gr (A.G.); abletsou@chem.uoa.gr (A.A.B.); dimdamalas@chem.uoa.gr (D.E.D.); ntho@chem.uoa.gr (N.S.T.); 2 Queensland Alliance for Environmental Health Sciences (QAEHS), The University of Queensland, Brisbane, QLD 4102, Australia; kevin.thomas@uq.edu.au; 3 Laboratory of Chemical Control of Pesticides, Scientific Directorate of Pesticides’ Control and Phytopharmacy, Benaki Phytopathological Institute, 8 St. Delta Street, 14561 Kifissia, Greece; n.maragou@bpi.gr

**Keywords:** high-resolution mass spectrometry, wastewater-based epidemiology, transformation products, human urinary metabolites, N, N-diethyl-meta-toluamide (DEET), 3-ethyl-carbamoyl benzoic acid, 3-phenoxybenzoic acid, captan, *cis*-1,2,3,6-tetrahydrophthalimide, biocides

## Abstract

Pesticides have been used in large amounts around the world for decades and are responsible for environmental pollution and various adverse effects on human health. Analysis of untreated wastewater can deliver useful information on pesticides’ use in a particular area and allow the assessment of human exposure to certain substances. A wide-scope screening method, based on liquid chromatography coupled to quadrupole-time-of-flight mass spectrometry, was applied, using both target and suspect screening methodologies. Daily composite influent wastewater samples were collected for seven or eight consecutive days in Athens between 2014 and 2020 and analyzed for 756 pesticides, their environmental transformation products and their human metabolites. Forty pesticides were quantified at mean concentrations up to 4.9 µg/L (tralkoxydim). The most abundant class was fungicides followed by herbicides, insect repellents, insecticides and plant growth regulators. In addition, pesticide transformation products and/or metabolites were detected with high frequency, indicating that research should be focused on them. Human exposure was evaluated using the wastewater-based epidemiology (WBE) approach and 3-ethyl-carbamoyl benzoic acid and *cis*-1,2,3,6-tetrahydrophthalimide were proposed as potential WBE biomarkers. Wastewater analysis revealed the presence of unapproved pesticides and indicated that there is an urgent need to include more transformation products in target databases.

## 1. Introduction

Pesticides are defined by the Food and Agricultural Organization (FAO) as substances or mixtures of substances intended for controlling, preventing, destroying, repelling or attracting any biological organism deemed to be a pest. The term “pesticides” is often used as a synonym for plant-protection products, which are used in agriculture to protect crops and in forestry to protect trees from pests and diseases. However, pesticides include also the class of biocides or biocidal products, whose field of application involves human and veterinary hygiene, food preservatives, antifouling products and preservatives among others [1]. Pesticides offer many benefits to the society, since they control pests, plant and human/livestock disease vectors and nuisance organisms and prevent organisms that damage structures [2]. However, the misuse of these chemicals leads to adverse consequences on human health (direct and indirect exposure) and the environment, which are numerous and well defined in the literature [3]. Consequently, there is a raised concern for their levels in the different environmental compartments. The concentrations of pesticides detected globally in various water systems have been found to range from nano- to micro-grams per liter [4,5,6,7,8].

Most pesticides are correlated to agricultural use and thus their occurrence has been mainly studied in ground and surface waters. Furthermore, their application in urban areas is critical and information on their presence in wastewater treatment plants (WWTPs) is of high interest. Pesticide mixtures are discharged into WWTPs from several sources, such as their use in industry, homes, public health, animals, the treatment of structures and the maintenance of green areas and water reserves [9,10]. 

A comprehensive study of all pesticides and their transformation products and/or metabolites (TP/M) in waters would be necessary to get a complete overview of this group of substances. However, thousands of pesticides are registered and this number is continuously increasing [11]. Therefore, it would be difficult to develop an analytical method to include all compounds. Indeed, most studies have used target methods based on a limited number of pesticides and TP/M [12,13,14]. Full-spectrum acquisition techniques could be a solution to this problem. Several research groups used high-resolution mass spectrometry (HRMS) techniques for the investigation of many pesticides and TP/M in different water samples applying target, suspect and non-target screening approaches [15,16,17,18,19,20]. 

The analysis of untreated influent wastewater (IWW) samples is very advantageous, since it provides data on pesticides’ use and can be applied for the evaluation of the general population’s exposure to specific pesticides in urban areas. Wastewater-based epidemiology (WBE) is currently used as a tool to assess the exposure to some pesticides by measurements of the corresponding human urinary metabolites [21]. So far, WBE has validated a few biomarkers for the estimation of the human exposure to pyrethroids, triazines and organophosphates [22,23]. This innovative approach has already been applied in more than twenty cities of ten European countries, covering millions of persons and a large number of substances [22,23,24,25,26,27]. 

The aim of the present work was to study the occurrence of a great number of pesticides, transformation products and human metabolites in untreated IWW in the urban area of Athens, Greece. Monitoring was performed in seven consecutive years (2014–2020) and the obtained data were used to investigate changes on pesticide use throughout this period and evaluate the human exposure. An HRMS wide-scope screening method based on liquid chromatography coupled to quadrupole-time-of-flight mass spectrometry (LC-QToF-MS) was applied for this purpose. Furthermore, WBE was used to assess potential health risks of certain pesticides to the general population.

## 2. Materials and Methods

### 2.1. Chemicals and Reagents

Information on analytical standards (742 parent pesticides and TP/M) is provided in Table S1. Methanol, acetonitrile, 2-propanol and ethyl acetate were of LC-MS grade and purchased from Merck (Darmstadt, Germany) and Fisher Scientific (Geel, Belgium). Sodium hydroxide for trace analysis, ammonium formate, ammonium acetate and formic acid were supplied by Fluka (Buchs, Switzerland). Distilled water was obtained by a Milli-Q purification apparatus (Millipore Direct-Q UV, Bedford, MA, USA). Materials for solid phase extraction (SPE) were purchased from Phenomenex (Torrance, CA, USA) and Biotage (Ystrad Mynach, UK) [20].

### 2.2. Wastewater Sampling

Daily composite IWW samples were collected from the WWTP of Athens, Greece. Flow-proportional samples were obtained for seven or eight consecutive days in March, from 2014 to 2020. The covered population is estimated approximately 4,000,000 inhabitants. Wastewater was sampled in high-density polyethylene bottles. 

### 2.3. Analytical Method

The analytical methodology is described in detail elsewhere [20]. Samples (100 mL) were filtered with glass fiber filters (pore size 0.7 μm; Millipore, Cork, Ireland), adjusted to pH 6.5 and extracted by an in-house SPE procedure. Elution was done in 4 mL of methanol/ethyl acetate (1:1, *v*/*v*) containing 2% ammonia, followed by 2 mL of methanol/ethyl acetate (1:1, *v*/*v*) containing 1.7% formic acid. Then, extracts were evaporated to a volume of 100 μL under a gentle nitrogen stream at room temperature and reconstituted to 0.5 mL with methanol/water (1:1, *v*/*v*). Finally, they were filtered through a regenerated cellulose filter (pore size 0.2 μm; Phenomenex, Torrance, CA, USA) and placed into glass vials for analysis. 

Analysis was performed by an LC system (Dionex UltiMate 3000 RSLC, Thermo Fisher Scientific, Am Kalkberg, Germany) interfaced to a QToF-MS (Maxis Impact, Bruker Daltonics, Bremen, Germany). The chromatographic column was thermostated at 30 °C and the injection volume was 5 µL. The mass spectrometric analysis was done using both ionization polarities and an electrospray ionization source was applied. The QToF MS operated in broadband collision-induced dissociation (bbCID) acquisition mode, recording spectra over the range *m*/*z* 50−1000 (scan rate of 2 Hz). This mode provided MS and MS/MS spectra at the same time, working at a low (4 eV) and a high (25 eV) collision energy. 

Data were analyzed by DataAnalysis 4.3, TargetAnalysis 1.3 and TASQ (Bruker Daltonics, Bremen, Germany). 

### 2.4. Quantification and Quality Assurance—Quality Control Measures

Samples were stored at −20 °C after collection at the laboratory of the WWTP. The samples were then transferred to a freezer in our laboratory and treated immediately to reduce the possible degradation of the analytes. Analysis was performed immediately or after a maximum of five days. Quantification of the detected compounds was done using standard addition calibration curves, freshly prepared, of the corresponding analytical standards. For compounds that the corresponding reference standards were not available in the lab (e.g., suspect list), a semi-quantification methodology was performed using other “similar” compounds. A 2D-based chemical similarity in-house model was applied to calculate the concentrations [28]. 

Quality assurance and quality control (QA/QC) measures are important to ensure consistency of result. Therefore, samples spiked with various standards at two different concentration levels were injected at the end of any analytical sequence and procedural blanks were included during the analysis. Furthermore, all samples were spiked with internal standards [28]. This process facilitated the evaluation of the system performance (e.g., reproducibility of the method) and corrected the obtained data for any undesired variation due to contamination, carry over, stability issues and extraction efficiency. 

### 2.5. Detection and Identification Criteria

Specific criteria were applied for the confirmation or identification of pesticides and their TP/M, including mass accuracy of the (de)protonated molecule (mass error < 5 ppm), accurate mass fragment ion(s) (mass error < 5 ppm) and retention time (±0.2 min from the reference standard). An in-house retention time prediction model was used to enhance the confidence level of the compounds identified by suspect analysis [29]. The criteria above were based on the confidence levels for small molecules [30]. 

### 2.6. Human Exposure

Untreated IWW is used to assess the population exposure (PES_intake_) to specific pesticides by the WBE approach. The mass loads of biomarkers (pesticide metabolites) are calculated by the measured concentration (Conc.) in IWW and the daily flow rate (F). Then, back-calculation of the human intake is performed considering the population size (P) and the correction factor (CF) according to the equation proposed by Rousis et al., 2017 [22]:

PES_intake_ = ((Conc. × F) × CF)/P

The exposure estimated by the equation above is used to evaluate the effects of the measured pesticides on human health. 

## 3. Results and Discussion

Urban wastewater contains numerous chemical compounds and a huge number of TP/M that could potentially pose risks to environmental and human health. A universal approach is therefore required to investigate all these compounds. SPE is considered as the main purification and pre-concentration technique in water analysis. Lately, different sorbents combined in one or more SPE column(s) have been used to extract numerous compounds with different physico-chemical properties [31]. Furthermore, advanced analytical techniques, such as LC and/or gas chromatography coupled to HRMS are used to get a comprehensive overview of organic contaminants in water systems [11,32,33]. Hence, this study used a general approach based on four different sorbents combined in one cartridge, as extraction procedure and an HRMS wide-scope screening method covering a very broad range of pesticides. 

### 3.1. Occurrence in Influent Wastewater

Pesticides were initially investigated using information obtained by reference standards, such as retention times and mass spectra. In total, forty pesticides and eight TP/M were detected in untreated IWW during the sampling period (2014–2020). These were fungicides (azoxystrobin, carbendazim, carboxin, climbazole, cyproconazole, cyprodinil, difenoconazole, fluconazole, fludioxonil, flutolanil, metalaxyl, penconazole, propamocarb, propiconazole, tebuconazole), herbicides (acetochlor, ametryn, amitrole, asulam, dinoterb, fluometuron, irgarol, metolachlor, napropamide, nicosulfuron, sethoxydim, terbacil, terbutryn, tralkoxydim), insect repellents (DEET, dimethyl phthalate, picaridin), insecticides (anabasine, dimethoate, fenamiphos, fipronil, pirimiphos methyl, thiamethoxam, thiodicarb), plant growth regulators (prohexadione) and TP/M (atrazine desethyl, atrazine desisopropyl, azoxystrobin acid, carbofuran-3-hydroxy, dimethachlor-ESA, metolachlor-morpholinon, propachlor-OXA, *cis*-1,2,3,6-tetrahydrophthalimide) (Tables S2–S8).

In addition, human urinary metabolites already validated as WBE biomarkers were investigated retrospectively by suspect analysis (Table 1). Metabolites of the most frequently detected pesticides were also investigated as potential WBE biomarkers (Table 1). The analysis was performed using information based on other studies (e.g., fragments and ionization mode) and retention times were estimated using an in-house prediction model [29]. A 2-min window was applied for the investigated compounds and three human metabolites were detected, namely 3-phenoxybenzoic acid, 3-ethyl-carbamoyl benzoic acid and metolachlor mercapturate.

**Figure 1:**
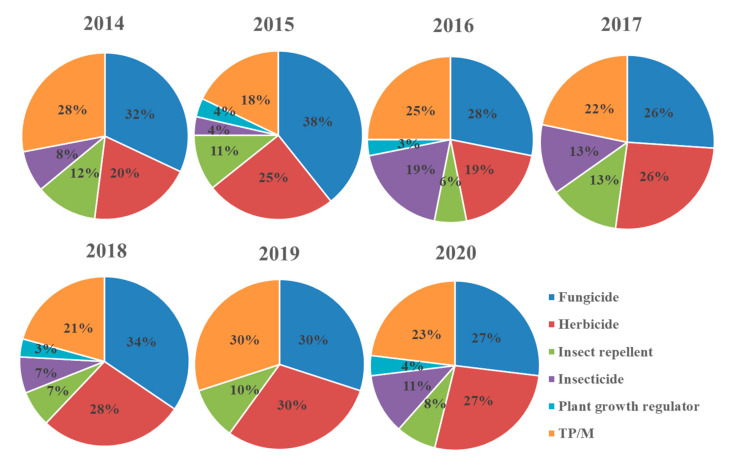
Detection frequencies of pesticide classes in influent wastewater sampled in Athens from 2014 to 2020.

The detection profile of pesticide classes in IWW remained stable throughout the sampling campaign and fungicides was the class most frequently detected, followed by herbicides, insect repellents, insecticides and plant growth regulators (Figure 1). These results were in accordance with the official sales data, expressed as Kg per year, provided by the Eurostat (https://ec.europa.eu/eurostat/web/agriculture/data/database, accessed on 2 September 2021). Fungicides and bactericides ranked first in sales for all years, except for the last two, whereas the same amounts were estimated for herbicides. Insecticides and acaricides ranked third, followed by other plant protection products and plant growth regulators. Furthermore, the presence of a high percentage of TP/M should be highlighted, which in some cases ranked second after fungicides. These data show that further research should be focused on TP/M.

Pesticide detection frequencies and concentrations are presented in Tables S2–S8 and Table 1. The most frequently detected compounds throughout the sampling period (detected from five to seven years) were azoxystrobin, carbendazim, climbazole, difenoconazole and fluconazole among fungicides; amitrole, fluometuron, metolachlor and tralkoxydim among herbicides; DEET and picaridin among insect repellents; and carbofuran-3-hydroxy, dimethachlor-ESA, *cis*-1,2,3,6-tetrahydrophthalimide and 3-ethyl-carbamoyl benzoic acid among TP/M.

The European Commission Water Framework Directive has established standards for individual and total pesticides at 0.1 μg/L and 0.5 μg/L respectively, in water monitoring [34]. Although, wastewater is not included in this regulation, pesticide concentrations were compared with these limits to investigate the pesticide toxicological profile of wastewater and better understand their extent of use. Most pesticides were quantified each year at concentrations below 100 ng/L (52%, 2016–76%, 2020), except from 2018 (32%). Some pesticides reached high mean concentrations, such as tralkoxydim (4.9 µg/L), metolachlor (4.8 µg/L), anabasine (3.6 µg/L), *cis*-1,2,3,6-tetrahydrophthalimide (3.3 µg/L), dimethachlor-ESA (2.5 µg/L), metolachlor-morpholinon (2.0 µg/L), sethoxydim (1.6 µg/L), carboxin (1.4 µg/L), terbacil (1.1 µg/L) and azoxystrobin (1.0 µg/L) far exceeding the individual limit. The total mean concentration of all pesticides was above the standard value of 0.5 µg/L for all years.

All the identified pesticides are summarized in Table S9 with their use and regulatory status under plant protection regulation (EC No 1107/2009), biocides regulation (EC No 528/2012) and REACH regulation (Registration, Evaluation, Authorisation and Restriction of Chemicals). REACH applies to all chemical substances; not only those used in industrial processes but also in our day-to-day lives, for instance in cleaning products, paints as well as in articles such as clothes, furniture and electrical appliances.

As already mentioned, pesticides are related to plant protection products for agricultural, domestic and forestry use and biocides have multiple fields of application, such as human and veterinary hygiene, food and wood preservatives and as antifouling products. Some of the detected substances have multiple uses, even outside the scope of pesticides, like in industry or laboratories, cosmetics and personal care products. Consequently, the concentration levels in the IWW of the city of Athens are the sum of all individual inputs occurring from the various uses. For instance, cyproconazole is approved as both plant protection and biocidal active substance, while the environmental occurrence of anabasine—which is not registered under any of the three regulations (plant protection, biocides and REACH)—can be related to tobacco products, based on literature data [35,36]. In addition, the detection of atrazine degradation products is attributed to the high atrazine application in the past and its high persistence in soils [37]. Furthermore, fluconazole is not registered under any of the three regulations, but it is known to be used as antifungal medicine, and climbazole is registered under REACH and used in cosmetics and personal care products.

Numerous pesticides and TP/M were investigated (Table S1) and 7% of them were identified in IWW. Even though this percentage could be considered low, some of the pesticides were used extensively, since they were detected in many years and, in some cases, at high concentrations. Moreover, the selected WWTP covers an urban area with few agricultural activities and, thus, the main sources of pesticides therein can be related to biocidal products, industries, homes, public areas etc.

The presence of some substances cannot be easily justified based on the available regulatory and use data. Acetochlor has been used in plant protection, but its approval has expired since 2011, while no other application is known. Likewise, the use of asulam sodium, dinoterb, sethoxydim, terbacil, terbutryn and the TP/M of carbofuran and propachlor are not approved. The presence of these substances should be further investigated and discussed with the participation of various bodies, such as scientists, policy makers, industry agents, farmers and representatives of local communities in order to achieve a sustainable management on their use. Strategies need to be developed with stakeholder involvement at all levels. National and/or local educational programs could inform all interested parties about water pollution and good practices regarding pesticide use in order to reduce the spread at its source. In addition, alternative pesticides that provide similar properties, but are less water soluble could replace harmful pesticides [34].

It must be pointed out that water pollution and ecosystem hazards (e.g., aquatic toxicity for fish, invertebrates and algae) cannot be assessed using untreated wastewater, as WWTPs implement several treatment processes that could alter these concentrations [20,38]. In the end, pesticides that are not eliminated by the WWTP end up in the Saronic Gulf, where they are further diluted [39].

#### 3.1.1. Fungicides

**Figure 2:**
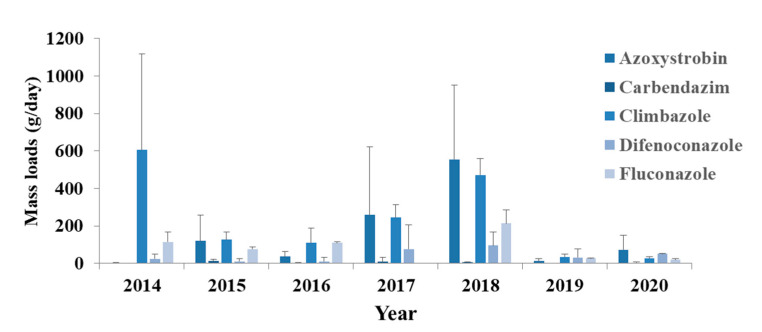
Mass loads of the most frequently detected fungicides (azoxystrobin, carbendazim, climbazole, difenoconazole and fluconazole) in influent wastewater collected in Athens.

Fifteen fungicides were identified in this study with only two (carbendazim and propiconazole) of them unapproved for use in Europe as active substances for plant-protection products. However, they are approved as biocides on preservation of films, woods and fibers and other uses on leather, rubber or polymers. Carbendazim is a fungicide itself and a metabolite of benomyl and thiophanate-methyl [40]; all of which are currently banned in Europe under EC 1107/2009. However, during the sampling period the use of thiophanate-methyl was approved. Carbendazim was detected in five out of seven sampling years (Figure 2) and its concentrations were low, with a maximum of 72 ng/L. It is one of the most detectable pesticides in water according to the literature [4,7,19,20,40,41,42,43], but it was complicated to evaluate the state of use due to its many sources. Propiconazole was found in only a few samples in 2016 at low concentrations. Thus, no concerns about environmental pollution are raised.

Eleven of the identified fungicides are approved for use in Europe and azoxystrobin (77%), cyprodinil (56%) and difenoconazole (52%) were among the most abundant (Figure 2). Their principal use is in agriculture and so they have been mainly investigated in water systems connected to farms or in areas where the agriculture activity is high [20,44,45,46,47]. Nevertheless, a part of the measured amounts is due to the use in horticulture, home and garden since the receiving wastewater in the investigated WWTP is mainly urban. Carboxin, cyproconazole, fludioxonil, flutolanil, metalaxyl, penconazole, propamocarb and tebuconazole had detection frequencies lower than 45%. Furthermore, their concentrations were low, except in the case of carboxin in 2019.

Climbazole (100%) and fluconazole (87%) were among the most frequently detected pesticides of this class (Figure 2) and they are both not included in the EU pesticides database. They are commonly used as antifungal agents in the treatment of human and animal fungal infections and have agricultural uses also. Nevertheless, their presence in urban wastewater, as in this study, is associated to domestic use, as they are widely used in personal care products, such as shampoos [48]. These compounds are usually found in environmental water samples as has been proved by several studies [49,50,51,52]. Some of these azolic compounds (e.g., fluconazole) present resistance to biodegradation and so their elimination through WWTPs is not efficient [49,50]. Thus, the negative removal and confirmed toxicity of these compounds in combination with their extended use, as it turned out, could cause damage to aquatic environment and actions must be taken.

#### 3.1.2. Herbicides

**Figure 3:**
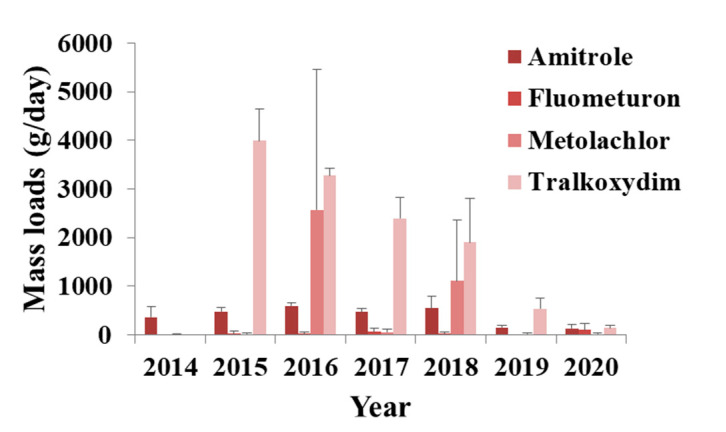
Mass loads of the most frequently detected herbicides (amitrole, fluometuron, metolachlor and tralkoxydim) in influent wastewater collected in Athens.

Many herbicides were identified, with most of them currently set to be banned in Europe under Reg 1107/2009. The use of only three pesticides (fluometuron, napropamide and nicosulfuron) is approved as plant protection active substances (Table S9). Amitrole, metolachlor and tralkoxydim were among the most frequently detected herbicides. Concentrations and mass loads for all three compounds were decreased significantly in the last two years of the sampling campaign (Figure 3). To the best of our knowledge no particular actions were undertaken by the authorities to eliminate these pesticides from the market during the last period. Therefore, no specific explanations can be given for this decline. However, potential reasons could be their absence on the stores and elimination of stocks. Amitrole and metolachlor were detected in many water samples before [4,7,17,19,20,42,53,54], but tralkoxydim is found in wastewater for the first time in the literature. Acetochlor, ametryn, asulam, dinoterb and terbutryn were found sporadically and mean concentrations ranged from 5 ng/L to 669 ng/L. Some of these compounds were also detected in various waters in other studies carried out in Italy, Spain, Greece, Germany, France, Switzerland, Denmark, Sweden, USA and China [17,19,20,41,43,53,55,56,57]. Terbacil was only detected in 2014 with mean concentration of 1135 ng/L. There is no cause for concern, since it was only detected once. This compound has not been widely studied and was also found in Spain [58]. Sethoxydim was detected in four different years presenting high concentrations and needs to be further investigated. No studies are available in the literature on the determination of sethoxydim in water.

Fluometuron was one of the most ubiquitous pesticides detected in this study (Figure 3). It belongs to phenylurea herbicides and is used in cotton. Fluometuron has been reported, by several studies, in various water types [20,54,59,60,61,62]. Cotton fields are not included in the area covered by the WWTP of Athens and thus the high presence of fluometuron may be due to non-agricultural uses, since this substance is registered under REACH and appears to have numerous uses (Table S9). Napropamide and nicosulfuron were both detected only in 2018. As the use of these pesticides is approved and the concentrations were low, no human health concerns are highlighted.

Irgarol is classified as herbicide [63] and is also included to the Biocidal Products Regulation (BPR, Regulation (EU) 528/2012), according to which its use is not approved. It was detected in 2015 only, at very low concentrations and thus there is no concern about its use. Irgarol is mainly applied as an antifouling agent and other uses, such as in construction and manufacturing have been reported [64].

#### 3.1.3. Insect Repellents

**Figure 4:**
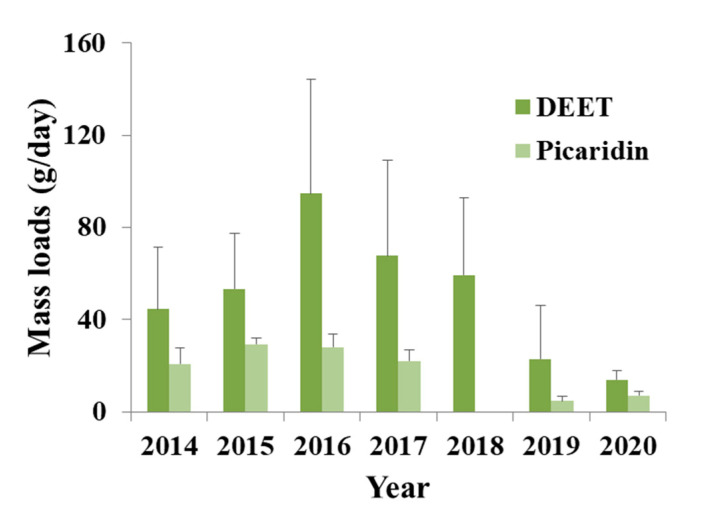
Mass loads of the most frequently detected insect repellents (N, N-diethyl-meta-toluamide (DEET) and picaridin) in influent wastewater collected in Athens.

Three insect repellents were found in the present study. DEET and picaridin are included to the Biocidal Products Regulation (BPR, Regulation (EU) 528/2012) and their use is approved (Table S9). DEET is one of the most frequently detected organic contaminants in waters, presenting a wide range of concentrations [65]. It was found in almost all samples (50/52) (Figure 4) and mean concentrations ranged from 17.9 ng/L (2020 sampling year) to 133.8 ng/L (2016 sampling year). Analysis of IWW has shown that this compound can be found in different concentrations, from traces (low ng/L level) to a few µg/L (e.g., [65,66,67,68,69]). Furthermore, seasonal and spatial variations have been detected [68]. Therefore, the lower concentrations found here, as compared with other works, could be explained by this variability, as sampling took place in March, a month with low application rates [68]. The main sources of this compound in untreated wastewater are assigned to domestic uses and disposal down the drain (individual and industrial) [65]. Although DEET is considered a ubiquitous compound, between little and no risk to human health is expected when applied according to product labels [70].

Picaridin is one of the most used synthetic repellents along with DEET and IR3535 and is less toxic than DEET [71]. It was detected in forty-two out of fifty-two samples and mean concentrations ranged from 6 ng/L (2019 sampling year) to 40 ng/L (2016 sampling year) (Figure 4). Picaridin has not been investigated as much as DEET in waters, and studies on its occurrence are limited [72,73,74,75]. Similarly, to DEET, low concentrations are related to the sampling period and higher amounts are expected during the main months of application, during summer.

Dimethyl phthalate was first used in the 1930s as an insect repellent [74] and since then its application field has expanded to include plastics, adhesives, coating agents, packaging, personal care products and others. It is a REACH registered substance. In this work the frequency of detection was 52% and mean concentrations reached 225 ng/L. In general, it is detected at various concentrations in untreated wastewater from relatively low (0.3 ng/L) to high (50 µg/L) values depending on the specific area [76,77]. The application of dimethyl phosphate as an insect repellent is currently limited and the found concentrations are probably due to its use as a plasticizer.

#### 3.1.4. Insecticides

A few insecticides were detected sporadically during the sampling periods. The use of five of them (thiamethoxam, fipronil, dimethoate, fenamiphos and thiodicarb) is not currently approved in Europe as active substances for plant protection, one (pirimiphos-methyl) is approved and anabasine is not included in the EU pesticides database (Table S9). Thiamethoxam was approved as wood preservative in the past (approval is expired) and currently is approved to control arthropods. It was detected in three years and is the only one of the detected pesticides that is included in the EU Watch List for emerging water pollutants [5]. Concentrations in IWW were low, but no information is available on the removal efficiency of conventional WWTPs to further assess the potential risk to the aquatic environment [78,79]. Fipronil is also approved as biocide for arthropods control, was detected more frequently (in four years) compared to the other insecticides and concentrations ranged 12–117 ng/L. The fipronil transformation products that are more stable and thus present higher environmental risk [80] were not detected. This compound has been systematically investigated due to its persistence during sewage treatment and toxicity to pollinators and aquatic invertebrates. It was identified in both wastewater and sludge in literature [7,81,82,83]. The presence of fipronil in urban wastewater was correlated to non-agricultural uses [81,84] and thus informing citizens about its harmful effects and ways to limit its use would be necessary. The detection frequencies were low for dimethoate (21%), fenamiphos (6%) and thiodicarb (19%) indicating that their use has been significantly restricted.

Pirimiphos-methyl was the only identified insecticide approved for use in Europe. It was found in two sampling years with concentrations lower than 15 ng/L. Therefore, no concern was raised about its use and its effects on public health.

Anabasine is a piperidine alkaloid and its use as insecticide has been confirmed. However, recent studies have shown that the presence of this compound in IWW is predominantly associated to tobacco use. Indeed, there are many studies that have used anabasine as a WBE biomarker to measure tobacco consumption [35,36]. Therefore, no further research was performed for anabasine, as the revealed concentrations were not related to its use as a pesticide and the estimation of tobacco consumption is outside the scope of the current study.

#### 3.1.5. Plant Growth Regulators

Prohexadione was the only plant growth regulator detected in the present study. It was found in four out of seven years with frequencies of detection ranging from 71% to 100% and mean concentrations 14.7–271 ng/L. Its use is approved in EU (Table S9) and is applied in fruits, vegetables and flowers [85,86,87,88,89]. The area covered by the WWTP of Athens is mainly urban and not much cultivation is presented. Thus, the occurrence of prohexadione in untreated wastewater is assigned to horticulture and transportation through contaminated fruits and vegetables (e.g., consumption and washing). To the best of our knowledge, this is the first study to confirm its presence in water systems.

#### 3.1.6. Transformation Products and/or Metabolites

Several TP/M were identified in the present work. Some of them were found sporadically (e.g., atrazine related products, metolachlor mercapturate and propachlor-OXA) and others at high detection frequencies. The compounds *cis*-1,2,3,6-tetrahydrophthalimide and dimethachlor-ESA were among those found at high concentrations, whereas the parent pesticides captan and dimethachlor respectively were not detected in any sample. The non-specific metabolite 3-PBA was detected in many samples, but none of its parent pyrethroids was found. Many of the corresponding pesticides were not detected and therefore, their negative occurrence may be due to degradation/metabolism and not to low application rates.

Transformation products and/or metabolites have not been monitored frequently in water systems, probably due to absence of reference standards and limited knowledge on their identity. However, a few studies have shown that these compounds could be presented at higher concentrations and can be more toxic compared to the parent pesticides [16,18]. Hence, it is needed to include more TP/M in the screening databases (target and/or suspect lists) to have a better view of pesticide occurrence in water.

### 3.2. Wastewater-Based Epidemiology and Human Exposure

Seven validated triazine, organophosphate and pyrethroid WBE biomarkers were investigated (Table 1) and only the 3-PBA metabolite was found. Detection was performed in negative mode, retention time was within the acceptable window range estimated by the prediction model and the mass errors of the precursor (213.0543) and product ions (169.0644 and 93.0346) were low. This compound is a metabolite of a large group of pyrethroid pesticides, including more than twenty compounds. Back-calculation was not performed, since the reference standard was not available in the lab, and none of the injected standards presented high (%) degree of similarity. The compound 3-PBA was not detected in the 2014, 2018 or 2020 sampling campaigns. The availability of the reference standard is required to estimate the exposure levels. In addition, other metabolites frequently found in untreated wastewater, such as 2-isopropyl-6-methyl-4-pyrimidinol, 3,5,6-trichloro-2-pyridinol and 3-(2,2-dichlorovinyl)-2,2-dimethyl-(1-cyclopropane) carboxylic acid [13,23,24,26] were not detected in the present study.

**Figure 5:**
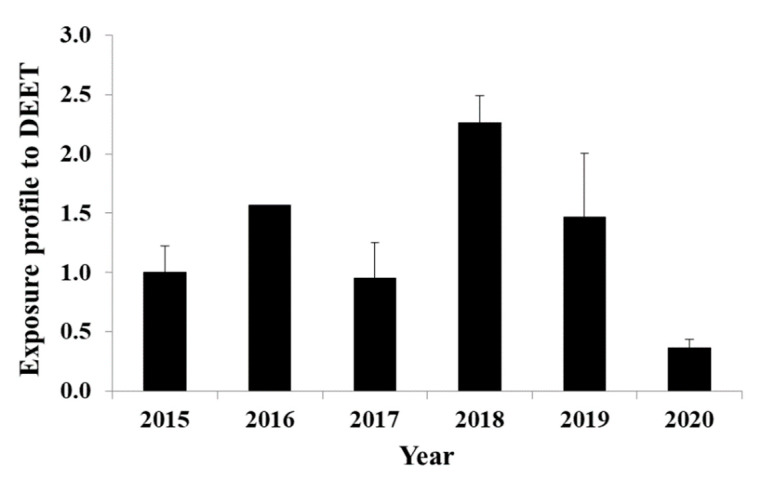
Exposure to N, N-diethyl-meta-toluamide (DEET) adjusted to 2015, back-calculated from 3-ethyl-carbamoyl benzoic acid concentrations in influent wastewater collected in Athens.

Nine human urinary metabolites of the most frequently detected pesticides were investigated (Table 1), and metolachlor mercapturate and 3-ethyl-carbamoyl benzoic acid were detected. Metolachlor mercapturate was detected in only two samples, and thus, no further investigation was performed. The compound 3-ethyl-carbamoyl benzoic acid was detected in positive mode, retention time was very close to the predicted one (within the accepted range) and mass error of the precursor (194.0811) and product ion (148.0757) was low; 3-ethyl-carbamoyl benzoic acid is a DEET metabolite and was found in twenty-five out of fifty-two samples. The presence of DEET in IWW has shown some discrepancies compared to application/usage data and thus, its suitability as WBE biomarker needs further analysis [68]. Therefore, in the present study, its specific metabolite 3-ethyl-carbamoyl benzoic acid is proposed as a WBE biomarker to assess the human exposure to DEET. Although, environmental degradation of DEET by microorganisms has been reported, none of the reported TPs was 3-ethyl-carbamoyl benzoic acid [65]. Thus, the presence of this metabolite in IWW could be attributed to human metabolism only, considering also the sources and routes of DEET in wastewater and environment [65]. Human exposure to DEET was estimated by 3-ethyl-carbamoyl benzoic acid measurements in IWW using pharmacokinetic data [90] and correction factors, as presented in Table 2. The only available human urinary metabolic data gave a range of excretion rate, and so two correction factors (13 and 3.88) were applied, using this range. Human exposure, using both CFs, ranged from 0.4–1.2 mg/day/person (2020) to 2.3–7.6 mg/day/person (2018) and the similarity to the quantification standard was low (40%). Consequently, the results in Figure 5 are adjusted according to year 2015 in order to investigate the time profile throughout the sampling campaign. The highest exposure was found in 2018 and the lowest in 2020. In general, the small variations could be described by different temperatures or other environmental conditions that affected the use of DEET by the population. The drop in 2020 was probably due to restrictions during the COVID-19 pandemic, such as staying indoors and store closures. The application of DEET is greatly affected by weather conditions, and, thus, more samples of the same month and probably from different months would be necessary to obtain an accurate exposure profile. The present work recommended the use of 3-ethyl-carbamoyl benzoic acid as a WBE biomarker to assess the human exposure to DEET and further research is required to fully validate this biomarker. New human urinary pharmacokinetic studies are mandatory to calculate the CF and in-lab, and/or in-sewer stability tests should be performed. One limitation of the current study was the non-availability of the reference standard, which did not permit the accurate determination of concentration levels.

The database included several TP/M, and eight of them were found in the samples. However, not all can be used to assess the human exposure to parent pesticides, as they do not meet all the requirements of a WBE biomarker [21]. Some compounds are referred to not only as human urinary metabolites, but as environmental transformation products, as well. The triazine metabolites, atrazine desethyl and atrazine desisopropyl, were found to be not suitable for WBE applications since their presence in IWW is greatly affected by sources other than human metabolism [23]. In addition, the degradation of the parent compounds in environmental media could be the source of the human metabolites azoxystrobin acid [92,93,94,95] and carbofuran-3-hydroxy [96,97,98] in IWW. The presence of metolachlor-morpholinon in the IWW could be attributed to various sources, such as human metabolism and environmental formation, and in the absence of available information to distinguish the actual source [99,100], no additional research was conducted. The dimethachlor-ESA compound is characterized as an environmental transformation product of dimethachlor [16]. Therefore, further research is suggested for these compounds, to evaluate their suitability as WBE biomarkers.

**Figure 6:**
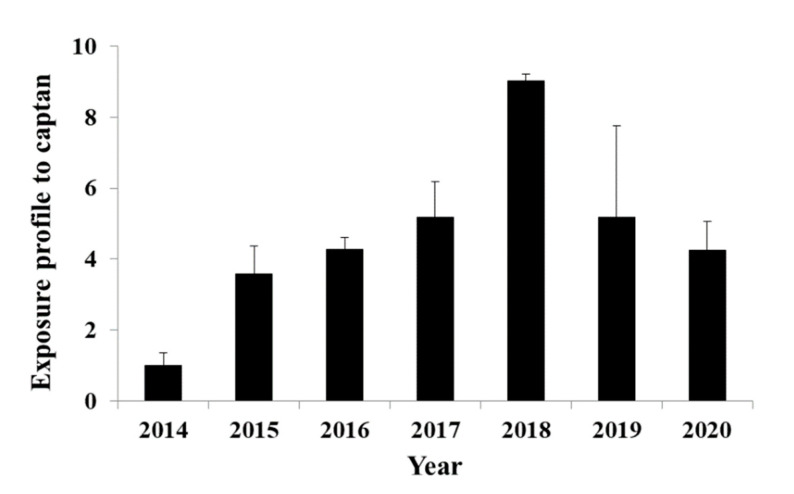
Captan exposure profile adjusted to 2014, back-calculated from *cis*-1,2,3,6-tetrahydrophthalimide concentrations in influent wastewater collected in Athens.

Captan is a commonly applied fungicide and human biomonitoring studies have used its specific metabolite *cis*-1,2,3,6-tetrahydrophthalimide to assess the human exposure [101,102,103]. This metabolite was detected in fifty out of the fifty-two tested samples (Tables S2–S8), unlike the parent pesticide, which was never detected. Human exposure to captan was estimated by its metabolite, using pharmacokinetic studies available in the literature [91] and the CF developed in the present work (Table 2). The estimated exposures ranged from 3.9 mg/day/person (2014) to 35.8 mg/day/person (2018). The above exposures were estimated semi-quantitatively, as the reference standard was not available and thus should be carefully evaluated. Thus, the results in Figure 6 are adjusted according to year 2014 to investigate the time profile throughout the sampling campaign. In 2018 the exposure was the highest, but no specific explanation could be given (Figure 6). More experiments are needed to validate this metabolite as a WBE biomarker, such as in-sample and wastewater stability and to verify that the measured amounts are not affected from other exogenic sources.

## 4. Conclusions

Untreated IWW were analyzed for 756 pesticides, transformation products and human urinary metabolites by LC-QToF-MS between 2014 and 2020 in Athens, Greece. Fifty-one pesticides and TP/M were detected belonging to various classes, such as fungicides, herbicides, insect repellents, insecticides and plant growth regulators. The use of many of the identified compounds is not approved in Europe and, thus, specific actions need to be taken to minimize their use.

The HRMS method investigated already validated WBE biomarkers retrospectively and 3-phenoxybenzoic acid was detected in four years. In addition, a few human metabolites of highly detected pesticides were searched and two of them were proposed as potential WBE biomarkers, namely 3-ethyl-carbamoyl benzoic acid and *cis*-1,2,3,6-tetrahydrophthalimide, also giving the corresponding correction factors. However, more experiments are needed to fully validate these biomarkers. A retention time prediction model was used to facilitate the identification of TP/M and a few false positive compounds were eliminated. In addition, a semi-quantification model was used to estimate the level of exposure when reference standards were not available in the laboratory. The HRMS method integrated with WBE was an effective tool for investigating a great number of pesticides and their TPs/Ms. Finally, this tool was able to assess the use and human exposure to these compounds.

## Figures and Tables

**Table 1:** Suspect analysis of human urinary metabolites in influent wastewater sampled in Athens between 2014 and 2020.

Human Urinary Metabolite	Parent Pesticide	Frequency of Detection (*n* = 52)	Retention Time (min.)	
			Sample	Predicted
**Validated Wastewater-Based Epidemiology Biomarkers (Metabolites)**				
atrazine mercapturate	atrazine	0		
3,5,6-trichloro-2-pyridinol	chlorpyrifos and chlorpyrifos-methyl	13	6.51	8.79
2-isopropyl-6-methyl-4-pyrimidinol	diazinon	14	1.70	4.94
malathion dicarboxylic acid	malathion	0		
malathion monocarboxylic acid	malathion	0		
3-phenoxybenzoic acid	group of pyrethroids	18	8.68	9.64
3-(2,2-dichlorovinyl)-2,2-dimethyl-(1-cyclopropane) carboxylic acid	permethrin, cypermethrin and cyfluthrin	28	2.65	8.80
**Potential Wastewater-Based Epidemiology Biomarkers (Metabolites)**				
methyl 5-hydroxy-2-benzimidazole carbamate	carbendazim	0		
3-diethyl-carbamoyl benzoic acid	N, N-diethyl-meta-toluamide	7	17.31	6.11
3-ethyl-carbamoyl benzoic acid	N, N-diethyl-meta-toluamide	25	4.30	4.69
N, N diethyl-3-hydroxymethylbenzamide	N, N-diethyl-meta-toluamide	23	2.77	5.72
fipronil sulfone	fipronil	0		
fipronil hydroxy	fipronil	0		
metolachlor mercapturate	metolachlor	2	7.34	8.92
clothianidin	thiamethoxam	0		

**Table 2:** Biomarkers to estimate human exposure to pesticides by influent wastewater analysis.

Metabolite	Parent Compound	Molar Mass Ratio	Excretion Rate (%)	Correction Factor (CF)
3-ethyl-carbamoyl benzoic acid	N, N-diethyl-meta-toluamide	0.99	7.6–25.5 [90]	13–3.88
*cis*-1,2,3,6-tetrahydrophthalimide	captan	1.99	3.5 [91]	56.9

## Supplementary Materials

The following are available online at https://www.mdpi.com/article/10.3390/toxics9100260/s1, Table S1: Analytical standards of parent pesticides, transformation products and metabolites. Table S2: Wastewater concentrations (ng/L), standard deviations (SD) and frequency of detection (DF) of pesticides and their transformation products and/or metabolites (TP/M) in influent wastewater in 2014 of Athens, Greece. Table S3: Wastewater concentrations (ng/L), standard deviations (SD) and frequency of detection (DF) of pesticides and their transformation products and/or metabolites (TP/M) in influent wastewater in 2015 of Athens, Greece. Table S4: Wastewater concentrations (ng/L), standard deviations (SD) and frequency of detection (DF) of pesticides and their transformation products and/or metabolites (TP/M) in influent wastewater in 2016 of Athens, Greece. Table S5: Wastewater concentrations (ng/L), standard deviations (SD) and frequency of detection (DF) of pesticides and their transformation products and/or metabolites (TP/M) in influent wastewater in 2017 of Athens, Greece. Table S6: Wastewater concentrations (ng/L), standard deviations (SD) and frequency of detection (DF) of pesticides and their transformation products and/or metabolites (TP/M) in influent wastewater in 2018 of Athens, Greece. Table S7: Wastewater concentrations (ng/L), standard deviations (SD) and frequency of detection (DF) of pesticides and their transformation products and/or metabolites (TP/M) in influent wastewater in 2019 of Athens, Greece. Table S8: Wastewater concentrations (ng/L), standard deviations (SD) and frequency of detection (DF) of pesticides and their transformation products and/or metabolites (TP/M) in influent wastewater in 2020 of Athens, Greece. Table S9: Pesticides determined from 2014 to 2020 in influent wastewater samples of Athens, Greece. Uses and regulatory status (https://ec.europa.eu/food/plant/pesticides/eu-pesticides-database/active-substances/, https://pesticidecompendium.bcpc.org, https://echa.europa.eu/, http://sitem.herts.ac.uk/aeru/iupac/index.htm, accessed on 2 September 2021).
