# Supplementary material for: Assessment of Environmental Pollution and Human Exposure to Pesticides by Wastewater Analysis in a Seven-Year Study in Athens, Greece
Source: Toxics. 2021 Oct 11;9(10):260. doi: 10.3390/toxics9100260 (PMC8537104; doi:10.3390/toxics9100260)
Supplement: Supplementary file 1 [file toxics-09-00260-s001.zip › toxics-1387278-supplementary.pdf]

toxics MDPI

Supplementary Materials:

Assessment of Environmental Pollution and Human Exposure to Pesticides by Wastewater Analysis in a Seven-Year Study in Athens, Greece

Nikolaos I. Rousis, Maria Denardou, Nikiforos Alygizakis, Aikaterini Galani, Anna A. Bletsou, Dimitrios E. Damalas, Niki C. Maragou, Kevin V. Thomas and Nikolaos S. Thomaidis

Table S1. Analytical standards of parent pesticides, transformation products and metabolites.

	Pesticide	Chemical formula
1	1,2,3,6-Tetrahydrophthalimide (cis-)	C8H9NO2
2	1,3,5(10)-estratrien-3 16' 17'-triol (E3 estriol)	C18H24O3
3	1-Naphthylacetic acid	C12H10O2
4	2,3,4,6-Tetrachlorophenol	C6H2Cl4O
5	2,4,5-T	C8H5Cl3O3
6	2,4,6-Trichlorophenol	C6H3Cl3O
7	2,4-D	C8H6Cl2O3
8	2,4-DB	C10H10Cl2O3
9	2,4-DB-methylester	C11H12Cl2O3
10	2,4-D-butylester	C12H14Cl2O3
11	2,4-Dimethylaniline (Metabolite 0f Amitraz)	C8H11N
12	2,4-D-methylester	C9H8Cl2O3
13	2-Aminobenzimidazole	C7H7N3
14	2-Benzyltetronic acid	C11H10O3
15	2-Methyl-4-amino-6-methoxy-s-triazine	C5H8N4O
16	2-Phenethylamine	C8H11N
17	2-Phenylphenol	C12H10O
18	3,4-(dichlorophenyl)-3-methyl urea	C8H8Cl2N2O
19	3,4-(dichlorophenyl)-urea	C7H6Cl2N2O
20	3,4-dichloroaniline	C6H5Cl2N
21	5-Chloro-2-methyl-4-isothiazolin-3-on (CMI)	C4H4ClNOS
22	Acephate	C4H10NO3PS
23	Acetamiprid	C10H11ClN4
24	Acetiamine	C16H22N4O4S
25	Acetochlor	C14H20ClNO2
26	Acetochlor-ESA	C14H21NO5S
27	Acetochlor-OXA	C14H19NO4
28	Acibenzolar-S-Methyl	C8H6N2OS2
29	Acifluorfen	C14H7ClF3NO5
30	Aclonifen	C12H9ClN2O3
31	Acrinathrin	C26H21F6NO5
32	Alachlor	C14H20ClNO2
33	Alachlor-ESA	C14H21NO5S
34	Alachlor-OXA	C14H19NO4
35	Alanycarb	C17H25N3O4S2
36	Albendazole	C12H15N3O2S
37	Albendazole sulfone	C12H15N3O4S
38	Aldicarb	C7H14N2O2S
39	Aldicarb-sulfone (Aldoxycarb)	C7H14N2O4S
40	Aldicarb-sulfoxide	C7H14N2O3S

41	Allethrin I	C19H26O3
42	Allethrin II	C19H26O3
43	Allidochlor	C8H12ClNO
44	Ametryn	C9H17N5S
45	Amidosulfuron	C9H15N5O7S2
46	Aminocarb (Metacil)	C11H16N2O2
47	Amitraz	C19H23N3
48	Amitrole	C2H4N4
49	Amorolfine	C21H35NO
50	Anabasine	C10H14N2
51	Ancymidol	C15H16N2O2
52	Anilazine. Dyrene	C9H5Cl3N4
53	Anilofos	C13H19ClNO3PS2
54	Aramite	C15H23ClO4S
55	Asana (Esfenvalerate)	C25H22ClNO3
56	Aspon	C12H28O5P2S2
57	Asulam	C8H10N2O4S
58	Atraton	C9H17N5O
59	Atrazin-desethyl-2-hydroxy (Prometon-Hydroxy-Desisopropyl)	C6H11N5O
60	Atrazine	C8H14ClN5
61	Atrazine-2-Hydroxy	C8H15N5O
62	Atrazine-desethyl	C6H10ClN5
63	Atrazine-desethyl-desisopropyl	C3H4ClN5
64	Atrazine-desisopropyl	C5H8ClN5
65	Atropine	C17H23NO3
66	AvermectinB1a (Abamectin)	C48H72O14
67	AvermectinB1b (Abamectin)	C47H70O14
68	Azaconazole	C12H11Cl2N3O2
69	Azadirachtin	C35H44O16
70	Azimsulfuron	C13H16N10O5S
71	Azinphos-ethyl	C12H16N3O3PS2
72	Azinphos-methyl (Guthion)	C10H12N3O3PS2
73	Aziprotryne	C7H11N7S
74	Azoxystrobin	C22H17N3O5
75	Azoxystrobin acid	C21H15N3O5
76	Barban	C11H9Cl2NO2
77	Beflubutamid	C18H17F4NO2
78	Benalaxyl	C20H23NO3
79	Benazolin	C9H6ClNO3S
80	Bendiocarb	C11H13NO4
81	Benfuracarb	C20H30N2O5S
82	Benodanil	C13H10INO
83	Benomyl	C14H18N4O3
84	Benoxacor	C11H11Cl2NO2
85	Bensulfuron-methyl	C16H18N4O7S
86	Bensulide	C14H24NO4PS3
87	Bensultap	C17H21NO4S4
88	Bentazone	C10H12N2O3S
89	Benthiavalicarb-isopropyl	C18H24FN3O3S
90	Benzethonium	C27H42NO2
91	Benzoic acid, 3-5-dibromo-4-hydroxy	C7H4Br2O3
92	Benzoximate	C18H18ClNO5
93	Benzoylprop-ethyl	C18H17Cl2NO3
94	Benzthiazuron	C9H9N3OS
95	Bifenazate	C17H20N2O3
96	Bifenox	C14H9Cl2NO5
97	Bifenox acid	C13H7Cl2NO5

98	Bifenthrin	C23H22ClF3O2
99	Bioallethrin	C19H26O3
100	Bioresmethrin	C22H26O3
101	Bispyribac	C19H18N4O8
102	Bitertanol	C20H23N3O2
103	Boscalid	C18H12Cl2N2O
104	Bromacil	C9H13BrN2O2
105	Bromadiolone	C30H23BrO4
106	Bromophos (Bromophos-methyl)	C8H8BrCl2O3PS
107	Bromophos-ethyl	C10H12BrCl2O3PS
108	Bromopropylate	C17H16Br2O3
109	Bromoxynil	C7H3Br2NO
110	Bromuconazole	C13H12BrCl2N3O
111	Bupirimate	C13H24N4O3S
112	Buprofezin (Z-isomer Buprofexin)	C16H23N3OS
113	Butachlor	C17H26ClNO2
114	Butafenacil	C20H18ClF3N2O6
115	Butamifos	C13H21N2O4PS
116	Butocarboxim-sulfoxid	C7H14N2O3S
117	Butoxycarboxim	C7H14N2O4S
118	Butralin	C14H21N3O4
119	Buturon	C12H13ClN2O
120	Butylate	C11H23NOS
121	Cadusafos	C10H23O2PS2
122	Cambendazol	C14H14N4O2S
123	Captafol	C10H9Cl4NO2S
124	Captan	C9H8Cl3NO2S
125	Carbamazine-Diethyl	C10H21N3O
126	Carbaryl	C12H11NO2
127	Carbendazim	C9H9N3O2
128	Carbetamide	C12H16N2O3
129	Carbofuran	C12H15NO3
130	Carbofuran-3-hydroxy	C12H15NO4
131	Carbophenothion	C11H16ClO2PS3
132	Carbosulfan	C20H32N2O3S
133	Carboxin	C12H13NO2S
134	Carfentrazone-ethyl	C15H14Cl2F3N3O3
135	CGA 321113 (Trifloxystrobin Metabolite)	C19H17F3N2O4
136	Chloramben	C7H5Cl2NO2
137	Chlorantraniliprole	C18H14BrCl2N5O2
138	Chlorbromuron	C9H10BrClN2O2
139	Chlorbufam	C11H10ClNO2
140	Chlorcyclizine	C18H21ClN2
141	Chlordimeform	C10H13ClN2
142	Chlorfenapyr	C15H11BrClF3N2O
143	Chlorfenprop-methyl	C10H10Cl2O2
144	Chlorfenson	C12H8Cl2O3S
145	Chlorfenvinphos (E/Z)	C12H14Cl3O4P
146	Chlorfluazuron	C20H9Cl3F5N3O3
147	Chloridazone	C10H8ClN3O
148	Chloridazone-methyl-desphenyl	C5H6ClN3O
149	Chlorimuronethyl	C15H15ClN4O6S
150	Chlormequat	C5H13ClN
151	Chlorobenzilate	C16H14Cl2O3
152	Chlorophacinone	C23H15ClO3
153	Chloropropylate	C17H16Cl2O3
154	Chlorothalonil-4-hydroxy	C8HN2OCl3

155	Chlorothiamid	C7H5Cl2NS
156	Chlorotoluron	C10H13ClN2O
157	Chloroxuron (Chloroxifenidim)	C15H15ClN2O2
158	Chlorpropham	C10H12ClNO2
159	Chlorpyriphos	C9H11Cl3NO3PS
160	Chlorpyriphos-methyl	C7H7Cl3NO3PS
161	Chlorsulfuron	C12H12ClN5O4S
162	Chlorthal-dimethyl (DCPA, Dacthal)	C10H6Cl4O4
163	Chlorthion	C8H9ClNO5PS
164	Chromafenozide	C24H30N2O3
165	Cinidon-ethyl	C19H17Cl2NO4
166	Cinosulfuron	C15H19N5O7S
167	Clethodim	C17H26ClNO3S
168	Climbazole	C15H17ClN2O2
169	Clodinafop-propargyl	C17H13ClFNO4
170	Clofentezine	C14H8Cl2N4
171	Clofibric acid	C10H11ClO3
172	Clomazone	C12H14ClNO2
173	Clopyralid	C6H3Cl2NO2
174	Clothiandin	C6H8ClN5O2S
175	Coumachlor	C19H15ClO4
176	Coumaphos	C14H16ClO5PS
177	Crimidine	C7H10ClN3
178	Crotamiton	C13H17NO
179	Crotoxyphos	C14H19O6P
180	Crufomate	C12H19ClNO3P
181	Cyanazine	C9H13ClN6
182	Cyanofenphos	C15H14NO2PS
183	Cyanophos	C9H10NO3PS
184	Cyazofamid	C13H13ClN4O2S
185	Cycloate	C11H21NOS
186	Cycloheximide	C15H23NO4
187	Cycloxydim	C17H27NO3S
188	Cycluron	C11H22N2O
189	Cyfluthrin (Baythroid)	C22H18Cl2FNO3
190	Cyhalothrin (lambda-)	C23H19ClF3NO3
191	Cymoxanil	C7H10N4O3
192	Cypermethrin	C22H19Cl2NO3
193	Cyprazin	C9H14ClN5
194	Cyproconazole	C15H18ClN3O
195	Cyprodinil	C14H15N3
196	Cyromazine	C6H10N6
197	Cythioate	C8H12NO5PS2
198	Daimuron (Dymron)	C17H20N2O
199	Dalapon	C3H4Cl2O2
200	Dazomet	C5H10N2S2
201	DDA (2.2-bis(4-chlorophenyl)-acetic acid)	C14H10Cl2O2
202	DEET (Diethyltoluamide)	C12H17NO
203	Deltamethrin	C22H19Br2NO3
204	Demeton-S	C8H19O3PS2
205	Demeton-S-methylsulfone	C6H15O5PS2
206	Demeton-S-methylsulfoxid (Oxydemeton-methyl)	C6H15O4PS2
207	Desmedipham	C16H16N2O4
208	Desmetryn	C8H15N5S
209	DET	C14H20N2
210	Diafenthiuron	C23H32N2OS
211	Dialifos	C14H17ClNO4PS2

212	Diallate	C10H17Cl2NOS
213	Diazinon	C12H21N2O3PS
214	Diazinon-O-analog	C12H21N2O4P
215	Dibutylchlorendate	C17H20Cl6O4
216	Dicamba	C8H6Cl2O3
217	Dicamba-methyl	C9H8Cl2O3
218	Dicapthon	C8H9ClNO5PS
219	Dichlofenthion	C10H13Cl2O3PS
220	Dichlofluanid	C9H11Cl2FN2O2S2
221	Dichlormid	C8H11Cl2NO
222	Dichlorobenzamide	Cl2C6H3CONH2
223	Dichlorophen	C13H10Cl2O2
224	Dichlorprop	C9H8Cl2O3
225	Dichlorprop-methyl	C10H10Cl2O3
226	Dichlorvos	C4H7Cl2O4P
227	Diclobutrazol	C15H19Cl2N3O
228	Diclofop	C15H12Cl2O4
229	Diclofop-methyl	C16H14Cl2O4
230	Dicloran	C6H4Cl2N2O2
231	Dicofol	C14H9Cl5O
232	Dicrotophos	C8H16NO5P
233	Diethofencarb	C14H21NO4
234	Difenoconazole	C19H17Cl2N3O3
235	Difenoxuron	C16H18N2O3
236	Difenzoquat	C17H17N2
237	Diflubenzuron	C14H9ClF2N2O2
238	Diflufenican	C19H11F5N2O2
239	Diflufenzopyr	C15H12F2N4O3
240	Diflufenzopyr	C18H24ClN3O
241	Dikegulac	C12H18O7
242	Dimefuron	C15H19ClN4O3
243	Dimethachlor	C13H18ClNO2
244	Dimethachlor-ESA	C13H19NO5S
245	Dimethachlor-OXA	C13H17NO4
246	Dimethenamid	C12H18ClNO2S
247	Dimethenamid-ESA	C12H19N1O5S2
248	Dimethenamid-OXA	C12H17NO4S
249	Dimethipin	C6H10O4S2
250	Dimethirimol	C11H19N3O
251	Dimethoate	C5H12NO3PS2
252	Dimethomorph	C21H22ClNO4
253	Dimethylanilin (N.N-)	C8H11N
254	Dimethyl-phthalate	C10H10O4
255	Dimethylvinphos	C10H10Cl3O4P
256	Dimetridazole	C5H7N3O2
257	Dimoxystrobin	C19H22N2O3
258	Dinex (2-Cyclohexyl-4.6-dinitrophenol)	C12H14N2O5
259	Diniconazole	C15H17Cl2N3O
260	Dinocap	C18H24N2O6
261	Dinoseb	C10H12N2O5
262	Dinotefuran	C7H14N4O3
263	Dinoterb	C10H12N2O5
264	Dioxacarb	C11H13NO4
265	Dioxathion	C12H26O6P2S4
266	Diphacinone	C23H16O3
267	Diphenamid	C16H17NO
268	Diphenylamine	C12H11N

269	Diquat	C12H12N2
270	Disulfoton	C8H19O2PS3
271	Disulfoton-sulfone	C8H19O4PS3
272	Disulfoton-sulfoxid	C8H19O3PS3
273	Ditalimfos	C12H14NO4PS
274	Dithiopyr	C15H16F5NO2S2
275	Diuron	C9H10Cl2N2O
276	DMSA (N N-Dimethylaminosulfanilid)	C8H12N2O2S
277	DNOC (4.6-dinitro-o-cresol)	C7H6N2O5
278	Dodemorph	C18H35NO
279	Dodine	C13H30N3
280	Drazoxolon	C10H8ClN3O2
281	Dyrene (Anilazine)	C9H5Cl3N4
282	Edifenphos	C14H15O2PS2
283	Emamectin B1a	C49H75NO13
284	Emamectin B1b	C48H73NO13
285	Endosulfan	C9H6Cl6O3S
286	Endosulfan-sulfate	C9H6Cl6O4S
287	Endothal	C8H10O5
288	EPN	C14H14NO4PS
289	Epoxiconazole	C17H13ClFN3O
290	EPTC	C9H19NOS
291	Esprocarb	C15H23NOS
292	Etaconazole	C14H15Cl2N3O2
293	Ethiofencarb	C11H15NO2S
294	Ethiofencarb-sulfone	C11H15NO4S
295	Ethiofencarb-sulfoxide	C11H15NO3S
296	Ethion	C9H22O4P2S4
297	Ethiprole	C13H9Cl2F3N4OS
298	Ethirimol	C11H19N3O
299	Ethofumesate	C13H18O5S
300	Ethoprop	C8H19O2PS2
301	Ethoprophos	C8H19O2PS2
302	Ethoxyquin	C14H19NO
303	Ethoxysulfuron	C15H18N4O7S
304	Etoxazole	C21H23F2NO2
305	Etrimfos	C10H17N2O4PS
306	Famoxadone	C22H18N2O4
307	Famphur	C10H16NO5PS2
308	Fenamidone	C17H17N3OS
309	Fenamiphos	C13H22NO3PS
310	Fenamiphos-sulfone	C13H22NO5PS
311	Fenarimol	C17H12Cl2N2O
312	Fenazaquin	C20H22N2O
313	Fenbuconazole	C19H17ClN4
314	Fenclofos (Ronnel)	C8H8Cl3O3PS
315	Fenfluoramine	C12H16F3N1
316	Fenfuram	C12H11NO2
317	Fenhexamid	C14H17Cl2NO2
318	Fenitrothion	C9H12NO5PS
319	Fenobucarb	C12H17NO2
320	Fenoprop (Silvex.2.4.5-TP)	C9H7Cl3O3
321	Fenoprop-methylester (Silvex-methyester)	C10H9Cl3O3
322	Fenothiocarb	C13H19NO2S
323	Fenoxaprop-P	C16H12ClNO5
324	Fenoxycarb	C17H19NO4
325	Fenpiclonil	C11H6Cl2N2

326	Fenpropathrin	C22H23NO3
327	Fenpropidin	C19H31N
328	Fenpropimorph	C20H33NO
329	Fenpyroximate	C24H27N3O4
330	Fenson	C12H9ClO3S
331	Fensulfothion	C11H17O4PS2
332	Fensulfothion-sulfon	C11H17O5PS2
333	Fenthion	C10H15O3PS2
334	Fenthion-oxon	C10H15O4PS
335	Fenthion-sulfon	C10H15O5PS2
336	Fenthion-sulfoxide	C10H15O4PS2
337	Triphenylstannylium	C18H15Sn
338	Fenuron	C9H12N2O
339	Fenvalerate	C25H22ClNO3
340	Fipronil	C12H4Cl2F6N4OS
341	Fipronil-desulfinyl	C12H4Cl2F6N4
342	Fipronil-sulfide	C12H4Cl2F6N4S
343	Fipronil-sulfone	C12H4Cl2F6N4O2S
344	Flamprop	C16H13ClFNO3
345	Flamprop-isopropyl	C19H19ClFNO3
346	Flazasulfuron	C13H12F3N5O5S
347	Flonicamid	C9H6F3N3O
348	Florasulam	C12H8F3N5O3S
349	Fluacrypyrim	C20H21F3N2O5
350	Fluazifop-p-butyl	C19H20F3NO4
351	Fluazinam	C13H4Cl2F6N4O4
352	Fluazuron	C20H10Cl2F5N3O3
353	Fluchloralin	C12H13ClF3N3O4
354	Fluconazole	C13H12F2N6O
355	Flucycloxuron	C25H20ClF2N3O3
356	Flucythrinate	C26H23F2NO4
357	Fludioxonil	C12H6F2N2O2
358	Flufenacet	C14H13F4N3O2S
359	Flufenacet-ESA	C11H14FNO4S
360	Flufenacet-OXA	C11H12FNO3
361	Flufenoxuron	C21H11ClF6N2O3
362	Flufenzine (Diflovidazin)	C14H7F2ClN4
363	Flumequine	C14H12FNO3
364	Flumethrin	C28H22Cl2FNO3
365	Flumetsulam	C12H9F2N5O2S
366	Flumioxazin	C19H15FN2O4
367	Fluometuron	C10H11F3N2O
368	Fluoroglycofen-ethyl	C18H13ClF3NO7
369	Fluoxastrobin	C21H16ClFN4O5
370	Fluquinconazole	C16H8Cl2FN5O
371	Fluridone	C19H14F3NO
372	Flurochloridone	C12H10Cl2F3NO
373	Fluroxypyr	C7H5Cl2FN2O3
374	Flurprimidol	C15H15F3N2O2
375	Flurtamone	C18H14F3NO2
376	Flusilazole	C16H15F2N3Si
377	Flutolanil	C17H16F3NO2
378	Flutriafol	C16H13F2N3O
379	Fluvalinate (tau-)	C26H22ClF3N2O3
380	Fomesafen	C15H10ClF3N2O6S
381	Fonofos	C10H15OPS2
382	Foramsulfuron	C17H20N6O7S

383	Forchlorfenuron	C12H10ClN3O
384	Formetanate	C11H15N3O2
385	Fosthiazate	C9H18NO3PS2
386	Fuberidazole	C11H8N2O
387	Furalaxyl	C17H19NO4
388	Furathiocarb	C18H26N2O5S
389	Furilazole	C11H13Cl2NO3
390	Glufosinate	C5H12NO4P
391	Griseofulvin	C17H17ClO6
392	Halfenprox	C24H23BrF2O3
393	Halofenozide	C18H19ClN2O2
394	Haloxyfop ethoxyethyl ester	C19H19ClF3NO5
395	Heliotrine	C16H27NO5
396	Heliotrine-N-oxide	C16H27NO6
397	Heptenophos	C9H12ClO4P
398	Hexaconazole	C14H17Cl2N3O
399	Hexaflumuron	C16H8Cl2F6N2O3
400	Hexazinone	C12H20N4O2
401	Hexythiazox	C17H21ClN2O2S
402	Imazalil	C14H14Cl2N2O
403	Imazamethabenz-methyl	C16H20N2O3
404	Imazamox	C15H19N3O4
405	Imazapyr	C13H15N3O3
406	Imazaquin	C17H17N3O3
407	Imazethapyr	C15H19N3O3
408	Imazosulfuron	C14H13ClN6O5S
409	Imibenconazole	C17H13Cl3N4S
410	Imidacloprid	C9H10ClN5O2
411	Imidacloprid-guanidine	C9H12Cl2N4
412	Imidacloprid-urea	C9H10ClN3O
413	Imidocarb	C19H20N6O
414	Inabenfide	C19H15ClN2O2
415	Inabenfide	C19H15ClN2O2
416	Indoxacarb	C22H17ClF3N3O7
417	Iodofenphos (Jodfenphos)	C8H8Cl2IO3PS
418	Ioxynil	C7H3I2NO
419	Iprobenfos	C13H21O3PS
420	Iprodione	C13H13Cl2N3O3
421	Iprovalicarb	C18H28N2O3
422	Irgarol	C11H19N5S
423	Irgarol-descyclopropyl	C8H15N5S
424	Isazophos	C9H17ClN3O3PS
425	Isocarbamid (Azolamide)	C8H15N3O2
426	Isocarbophos	C11H16NO4PS
427	Isoconazole	C18H14Cl4N2O
428	Isofenphos	C15H24NO4PS
429	Isofenphos-methyl	C14H22NO4PS
430	Isoprocarb	C11H15NO2
431	Isopropalin	C15H23N3O4
432	Isoproturon	C12H18N2O
433	Isoproturon-didemethyl (1-(4-Isoprophenyl)urea)	C10H14N2O
434	Isoxaben	C18H24N2O4
435	Isoxadifen-ethyl	C18H17NO3
436	Isoxaflutole	C15H12F3NO4S
437	Isoxathion	C13H16NO4PS
438	Ivermectin	C48H74O14
439	Kresoxim-methyl	C18H19NO4

440	Lactofen	C19H15ClF3NO7
441	Lenacil	C13H18N2O2
442	Leptophos	C13H10BrCl2O2PS
443	Lethane 384	C9H17NO2S
444	Linuron	C9H10Cl2N2O2
445	Lufenuron	C17H8Cl2F8N2O3
446	Malaoxon	C10H19O7PS
447	Malathion	C10H19O6P1S2
448	MCPA	C9H9ClO3
449	MCPB	C11H13ClO3
450	Mecarbam	C10H20NO5PS2
451	Mecoprop	C10H11ClO3
452	Mecoprop-methylester	C11H13ClO3
453	Mefenacet	C16H14N2O2S
454	Mefenpyr-diethyl	C16H18Cl2N2O4
455	Mefluidide	C11H13F3N2O3S
456	Melamine	C3H6N6
457	Mepanipyrim	C14H13N3
458	Mepronil	C17H19NO2
459	Mercaptobenzothiazole	C7H5NS2
460	Mesotrion-MNBA	C8H7NO6S
461	Metalaxyl	C15H21NO4
462	Metamitron	C10H10N4O
463	Metaxalone	C12H15NO3
464	Metazachlor	C14H16ClN3O
465	Metazachlor-ESA	C14H17N3O4S
466	Metazachlor-OXA	C14H15N3O3
467	Metconazole	C17H22ClN3O
468	Methabenzthiazuron	C10H11N3OS
469	Methacrifos	C7H13O5PS
470	Methamidophos	C2H8NO2PS
471	Methfuroxam	C14H15NO2
472	Methidathion	C6H11N2O4PS3
473	Methiocarb (Mercaptodimethur)	C11H15NO2S
474	Methiocarb-sulfone	C11H15NO4S
475	Methiocarb-sulfoxide	C11H15NO3S
476	Methomyl	C5H10N2O2S
477	Methoprene	C19H34O3
478	Methoprotryne	C11H21N5OS
479	Methoxyfenozide	C22H28N2O3
480	Methyl 2-dimethoxyphosphinothioylsulfanylacetate	C5H11O4PS2
481	Metobromuron	C9H11BrN2O2
482	Metolachlor	C15H22ClNO2
483	Metolachlor-ESA	C15H23NO5S
484	Metolachlor-morpholinon	C14H19NO2
485	Metolachlor-OXA	C15H21NO4
486	Metolcarb	C9H11NO2
487	Metominostrobin (E-Isomer)	C16H16N2O3
488	Metominostrobin (Z-Isomer)	C16H16N2O3
489	Metosulam	C14H13Cl2N5O4S
490	Metoxuron	C10H13ClN2O2
491	Metrafenone	C19H21BrO5

492	Metribuzin	C8H14N4OS
493	Metribuzin-Desamino (DA)	C8H13N3OS
494	Metribuzin-Diketo (DK)	C7H12N4O2
495	Metsulfuron-methyl	C14H15N5O6S
496	Mevinphos	C7H13O6P
497	Mexacarbate	C12H18N2O2
498	MGK-264	C17H25NO2
499	Molinate	C9H17NOS
500	Monocrotaline	C16H23NO6
501	Monocrotaline-N-oxide	C16H23NO7
502	Monocrotophos	C7H14NO5P
503	Monolinuron	C9H11ClN2O2
504	Monuron	C9H11ClN2O
505	Morpholinon	C14H19N1O2
506	Myclobutanil	C15H17ClN4
507	N'-(2 4-Dimethylphenyl)-N-methylformamidine	C10H14N2
508	N.N-Dimethyl-N'-p-tolylsulphamide (DMST)	C9H14N2O2S
509	N-2-4-Dimethylphenylformamide (DMF, Amitraz Metabolite)	C9H11NO
510	Naled	C4H7Br2Cl2O4P
511	Naphthoxyaceticacid (beta-)	C12H10O2
512	Napropamide	C17H21NO2
513	Naptalam (N-1-Naphthylphthalamicacid)	C18H13NO3
514	Neburon	C12H16Cl2N2O
515	Niclosamide	C13H8Cl2N2O4
516	Nicosulfuron	C15H18N6O6S
517	Nitenpyram	C11H15ClN4O2
518	Nitrofen	C12H7Cl2NO3
519	Nitrothal-isopropyl	C14H17NO6
520	Norflurazon	C12H9ClF3N3O
521	Novaluron	C17H9ClF8N2O4
522	Noviflumuron	C17H7Cl2F9N2O3
523	Nuarimol	C17H12ClFN2O
524	o,o,o-Triethylphosphorothioate	C6H15O3PS
525	Ofurace	C14H16ClNO3
526	Omethoate	C5H12NO4PS
527	Orbencarb	C12H16ClNOS
528	Oryzalin	C12H18N4O6S
529	Oxadiargyl	C15H14Cl2N2O3
530	Oxadiazon	C15H18Cl2N2O3
531	Oxadixyl	C14H18N2O4
532	Oxamyl	C7H13N3O3S
533	Oxasulfuron	C17H18N4O6S
534	Oxfendazole	C15H13N3O3S
535	Oxybutynin	C22H31NO3
536	Oxycarboxin	C12H13NO4S
537	Oxydemeton-methyl	C6H15O4PS2
538	Oxyfluorfen	C15H11ClF3NO4
539	p,p-Dichlorobenzophenone	C13H8Cl2O
540	Paclobutrazole	C15H20ClN3O
541	Paraoxon	C10H14NO6P
542	Paraoxon-methyl	C8H10NO6P

543	Parathion	C10H14NO5PS
544	Parathion-methyl	C8H10NO5PS
545	PCP	C6HCl5O
546	Pebulate	C10H21NOS
547	Penconazole	C13H15Cl2N3
548	Pencycuron	C19H21ClN2O
549	Pendimethalin	C13H19N3O4
550	Penfluron	C15H9F5N2O2
551	Pentanochlor	C13H18ClNO
552	Permethrin	C21H20Cl2O3
553	Pethoxamid	C16H22ClNO2
554	Phenmedipham	C16H16N2O4
555	Phenothrin (tech)	C23H26O3
556	Phenthoate	C12H17O4PS2
557	Phorate	C7H17O2PS3
558	Phorate-oxon	C7H17O3PS2
559	Phosalone	C12H15ClNO4PS2
560	Phosmet	C11H12NO4PS2
561	Phosphamidon (Dimecron)	C10H19ClNO5P
562	Phoxim	C12H15N2O3PS
563	P-Hydroxymesocarb	C18H18N4O3
564	Picaridin (Icaridin)	C12H23NO3
565	Picloram	C6H3Cl3N2O2
566	Picolinafen	C19H12F4N2O2
567	Piperazine	C4H10N2
568	Piperonylbutoxide	C19H30O5
569	Pirimicarb	C11H18N4O2
570	Pirimicarb-desmethyl	C10H16N4O2
571	Pirimiphos-ethyl	C13H24N3O3PS
572	Pirimiphos-methyl	C11H20N3O3PS
573	Pretilachlor	C17H26ClNO2
574	Prochloraz	C15H16Cl3N3O2
575	Procymidone	C13H11Cl2NO2
576	Profenophos	C11H15BrClO3PS
577	Profoxydim	C24H32ClNO4S
578	Proguanil	C11H16N5Cl
579	Prohexadione	C10H12O5
580	Promecarb	C12H17NO2
581	Prometon	C10H19N5O
582	Prometryn	C10H19N5S
583	Propachlor	C11H14ClNO
584	Propachlor-ESA	C11H15NO4S
585	Propachlor-OXA	C11H13NO3
586	Propamocarb	C9H20N2O2
587	Propanil	C9H9Cl2NO
588	Propaphos	C13H21O4PS
589	Propaquizafop	C22H22ClN3O5
590	Propargite	C19H26O4S
591	Propazine	C9H16ClN5
592	Propazine-2-hydroxy (Prometon-Hydroxy)	C9H17N5O
593	Propetamphos	C10H20NO4PS

594	Propham	C10H13NO2
595	Propiconazole	C15H17Cl2N3O2
596	Propoxur	C11H15NO3
597	Propyzamide (Pronamide)	C12H11Cl2NO
598	Proquinazid	C14H17IN2O2
599	Prosulfocarb	C14H21NOS
600	Prosulfuron	C15H16F3N5O4S
601	Prothioconazole	C14H15Cl2N3OS
602	Prothioconazole-desethio	C14H15Cl2N3O
603	Pymetrozine	C10H11N5O
604	Pyraclostrobin	C19H18ClN3O4
605	Pyraflufen-ethyl	C15H13Cl2F3N2O4
606	Pyrazophos	C14H20N3O5PS
607	Pyrazoxyfen	C20H16Cl2N2O3
608	Pyrethrin	C21H28O3
609	Pyrethrins: Cinerin	C20H28O3
610	Pyrethrins: Jasmolin	C21H30O3
611	Pyributicarb	C18H22N2O2S
612	Pyridaben	C19H25ClN2OS
613	Pyridaphenthion	C14H17N2O4PS
614	Pyridate	C19H23ClN2O2S
615	Pyrifenox	C14H12Cl2N2O
616	Pyrimethanil	C12H13N3
617	Pyrimidifen	C20H28ClN3O2
618	Pyriproxyfen	C20H19NO3
619	Quinalphos	C12H15N2O3PS
620	Quinclorac	C10H5Cl2NO2
621	Quinmerac	C11H8ClNO2
622	Quinoxyphen	C15H8Cl2FNO
623	Quizalofop	C17H13ClN2O4
624	Quizalofop-ethyl	C19H17ClN2O4
625	Rabenzazole	C12H12N4
626	Resmethrin	C22H26O3
627	Resmethrin	C22H26O3
628	Retrorsine	C18H25NO6
629	Retrorsine-N-oxide	C18H25NO7
630	Rimsulfuron	C14H17N5O7S2
631	Rotenone	C23H22O6
632	Schradan	C8H24N4O3P2
633	Sebuthylazine	C9H16ClN5
634	Secbumeton	C10H19N5O
635	Senecionine	C18H25NO5
636	Senecionine-N-oxide	C18H25NO6
637	Senkirkine	C19H27NO6
638	Sethoxydim	C17H29NO3S
639	Siduron	C14H20N2O
640	Simazine	C7H12ClN5
641	Simazine 2-Hydroxy	C7H13N5O
642	Simetryn	C8H15N5S
643	Spinosad A (Spinosyn A)	C41H65NO10
644	Spinosad D	C42H67NO10

645	Spinosyn B or K	C40H63NO10
646	Spirodiclofen	C21H24Cl2O4
647	Spiromesifen	C23H30O4
648	Spiroxamine	C18H35NO2
649	Strychnine	C21H22N2O2
650	Sulcotrione	C14H13ClO5S
651	Sulfometuron-methyl	C15H16N4O5S
652	Sulfotepp	C8H20O5P2S2
653	Sulprofos (Bolstar)	C12H19O2PS3
654	SWEP.MCC	C8H7Cl2NO2
655	TCMTB	C9H6N2S3
656	Tebuconazole	C16H22ClN3O
657	Tebufenozide	C22H28N2O2
658	Tebupirimphos	C13H23N2O3PS
659	Tebutame	C15H23NO
660	Tebuthiuron	C9H16N4OS
661	Teflubenzuron	C14H6Cl2F4N2O2
662	Tefluthrin	C17H14ClF7O2
663	Temephos	C16H20O6P2S3
664	TEPP	C8H20O7P2
665	Tepraloxydim	C17H24ClNO4
666	Terbacil	C9H13ClN2O2
667	Terbufos	C9H21O2PS3
668	Terbufos-sulfone	C9H21O4PS3
669	Terbufos-sulfoxide	C9H21O3PS3
670	Terbumeton	C10H19N5O
671	Terbuthylazine	C9H16ClN5
672	Terbuthylazine-2-hydroxy	C9H17N5O
673	Terbuthylazine-desethyl	C7H12ClN5
674	Terbuthylazine-desethyl-2-hydroxy	C7H13N5O
675	Terbutryn	C10H19N5S
676	Tetrachlorvinphos (Stirofos)	C10H9Cl4O4P
677	Tetraconazole	C13H11Cl2F4N3O
678	Tetradifon	C12H6Cl4O2S
679	Tetramethrin	C19H25NO4
680	Thenylchlor	C16H18ClNO2S
681	Thiabendazole	C10H7N3S
682	Thiacloprid	C10H9ClN4S
683	Thiacloprid-amide	C10H11ClN4OS
684	Thiamethoxam	C8H10ClN5O3S
685	Thiazopyr	C16H17F5N2O2S
686	Thidiazuron	C9H8N4OS
687	Thifensulfuron-methyl	C12H13N5O6S2
688	Thiobencarb	C12H16ClNOS
689	Thiocyclam	C5H11NS3
690	Thiodicarb	C10H18N4O4S3
691	Thiofanox	C9H18N2O2S
692	Thiometon	C6H15O2PS3
693	Thionazin (Zinophos)	C8H13N2O3PS
694	Thiophanate-methyl	C12H14N4O4S2
695	Thiophanat-ethyl	C14H18N4O4S2

696	Thiram (Tetramethylthiuramdisulfide, TMTD)	C6H12N2S4
697	Tinidazole	C8H13N3O4S
698	Tiocarbazil	C16H25NOS
699	Tokuthion (Prothiophos)	C11H15Cl2O2PS2
700	Tolclofos-methyl	C9H11Cl2O3PS
701	Tolfenpyrad	C21H22ClN3O2
702	Tolnaftate	C19H17NOS
703	Tolyfluanid	C10H13Cl2FN2O2S2
704	Tralkoxydim	C20H27NO3
705	Tralomethrin	C22H19Br4NO3
706	Tranexamic acid	C8H15NO2
707	Triadimefon	C14H16ClN3O2
708	Triadimenol	C14H18ClN3O2
709	Triallate	C10H16Cl3NOS
710	Triasulfuron	C14H16ClN5O5S
711	Triazamate	C13H22N4O3S
712	Triazophos	C12H16N3O3PS
713	Triazoxide	C10H6ClN5O
714	Tribenuron-methyl	C15H17N5O6S
715	Tribufos (Merphos oxide. DEF)	C12H27OPS3
716	Trichlorfon (Dylox)	C4H8Cl3O4P
717	Trichloronate	C10H12Cl3O2PS
718	Triclabendazole	C14H9Cl3N2OS
719	Triclocarban	C13H9Cl3N2O
720	Triclopyr	C7H4Cl3NO3
721	Triclopyr-methylester	C8H6Cl3NO3
722	Tricyclazole	C9H7N3S
723	Trietazine	C9H16ClN5
724	Trifloxystrobin	C20H19F3N2O4
725	Trifloxysulfuron	C14H14F3N5O6S
726	Triflumizole	C15H15ClF3N3O
727	Triflumuron	C15H10ClF3N2O3
728	Triflusulfuron-methyl	C17H19F3N6O6S
729	Triforine	C10H14Cl6N4O2
730	Trimethacarb (2,3,5-)	C11H15NO2
731	Trimethacarb (3,4,5-)	C11H15NO2
732	Trinexapac acid	C11H12O5
733	Trinexapac-ethyl	C13H16O5
734	Triphenylphosphate	C18H15O4P
735	Triticonazole	C17H20ClN3O
736	Uniconazole	C15H18ClN3O
737	Vamidothion	C8H18NO4PS2
738	Vegadex (Sulfallate)	C8H14ClNS2
739	Vernolate	C10H21NOS
740	Warfarin	C19H16O4
741	XMC	C10H13NO2
742	Zoxamide	C14H16Cl3NO2

Table S2. Wastewater concentrations (ng/L), standard deviations (SD) and frequency of detection (DF) of pesticides and their transformation products and/or metabolites (TP/M) in influent wastewater in **2014** of Athens, Greece.

Analyte	Polarity	Mean	SD	Median	Min.	Max.	DF	Pesticide class
Ametryn	+	9.2	1.1	10	8.3	11.3	8/8	herbicide
Amitrole	+	554	21	524	351	973	7/8	herbicide
Atrazine-desethyl	+	176	16	166	126	275	8/8	TP/M
Atrazine-desisopropyl	+	217	17	196	164	313	5/8	TP/M
Azoxystrobin	+	7.04	0.38	7	3.83	11.07	4/8	fungicide
Azoxystrobin acid	+	72.0	46.0	74.5	13	127	8/8	TP/M
Carbofuran-3-hydroxy	+	19.8	3.0	20	16	22	8/8	TP/M
Climbazole	+	790	670	800	150	2000	8/8	fungicide
DEET (Diethyltoluamide)	+	58.1	35.0	55.2	25.0	110	8/8	insect repellent
Difenoconazole	+	31.2	33	32.1	5.0	82.1	5/8	fungicide
Dimethachlor-ESA	+	2500	650	2440	1800	3700	8/8	TP/M
Dimethoate	+	70	56	72	32	150	4/8	insecticide, acaricide
Fluconazole	+	150	70	155	88	260	8/8	fungicide
Flutolanil	+	6.1	2.0	7.1	3.0	9.0	8/8	fungicide
Metalaxyl	+	76.0	12.0	77.1	3.0	320	6/8	fungicide
Metolachlor	+	13.1	13	13.0	1	28	8/8	herbicide
Metolachlor-morpholinon	+	12.62	0.31	14	8.81	18.2	7/8	TP/M
Penconazole	+	33.1	34.0	35.0	4.0	100	8/8	fungicide
Phthalate-dimethyl	+	43.8	3.8	40	33.9	64.4	8/8	insect repellent
Picaridin (Icaridin)	+	27.1	9.0	25.6	20.1	43.2	5/8	insect repellent
Terbacil	+	1135	111	1156	967	1282	8/8	herbicide
Dinoterb	-	23	25	24	5	73	8/8	herbicide
Fipronil	-	12	4.0	11.3	7	19	8/8	insecticide
Fludioxonil	-	6.2	6.0	6.5	2.0	20.1	8/8	fungicide
cis-1,2,3,6-Tetrahydrophthalimide	-	3379	358	3695	1309	4438	8/8	TP/M

Table S3. Wastewater concentrations (ng/L), standard deviations (SD) and frequency of detection (DF) of pesticides and their transformation products and/or metabolites (TP/M) in influent wastewater in **2015** of Athens, Greece.

Analyte	Polarity	Mean	SD	Median	Min.	Max.	DF	Pesticide class
Amitrole	+	588	61	589	483	689	8/8	herbicide
Anabasine	+	2338	157	2385	1873	2684	8/8	insecticide
Azoxystrobin	+	178	22	68	17	478	7/8	fungicide
Azoxystrobin acid	+	85.4	7.3	85.1	25.2	169.0	5/8	TP/M
Carbendazim	+	16.0	9.1	14.5	10	34	8/8	fungicide
Carbofuran-3-hydroxy	+	48.8	4.0	48.5	40.6	57.7	8/8	TP/M
Carboxin	+	7.52	0.90	7.56	3.67	9.17	8/8	fungicide
Climbazole	+	159	17	137	102	271	8/8	fungicide
Cyproconazole	+	351.1					1/8	fungicide
Cyprodinil	+	7.2	1.1	7.7	3.6	10.8	8/8	fungicide
DEET (Diethyltoluamide)	+	64.3	5.8	55.4	42.8	112.8	8/8	insect repellent
Difenoconazole	+	37.9	5.7	32.3	24.3	57.1	3/8	fungicide
Fluconazole	+	95	14	95	65	124	8/8	fungicide
Fluometuron	+	99	11	39	24	234	3/8	herbicide
Flutolanil	+	71.3	6.5	71.0	55.2	83.5	5/8	fungicide
Irgarol	+	2.58	0.27	2.70	1.60	3.11	8/8	herbicide
Metalaxyl	+	22.46	0.56	17.61	16.82	32.95	3/8	fungicide
Metolachlor	+	27.2	2.6	13.3	8.4	89.1	7/8	herbicide
Phthalate-dimethyl	+	41.7	3.2	37.7	21.2	76.0	7/8	insect repellent
Picaridin (Icaridin)	+	36.2	3.3	35.9	27.6	45.1	8/8	insect repellent
Prohexadione	+	121	18	124	30	185	6/8	plant growth regulator
Sethoxydim	+	1089	76	1109	860	1233	5/8	herbicide
Tralkoxydim	+	4885	630	4789	3782	6006	8/8	herbicide
Dinoterb	-	26.4	1.2	26.4	25.5	27.3	2/8	herbicide
cis-1,2,3,6-Tetrahydrophthalimide	-	1256	118	1280	788	1760	8/8	TP/M

Table S4. Wastewater concentrations (ng/L), standard deviations (SD) and frequency of detection (DF) of pesticides and their transformation products and/or metabolites (TP/M) in influent wastewater in **2016** of Athens, Greece.

Analyte	Polarity	Mean	SD	Median	Min.	Max.	DF	Pesticide class
Amitrole	+	849	85	849	706	976	8/8	herbicide
Anabasine	+	3641	317	3430	2588	5655	8/8	insecticide
Asulam	+	669	64	696	455	790	6/8	herbicide
Azoxystrobin	+	71.1	5.0	75.8	39.8	107.5	6/8	fungicide
Azoxystrobin acid	+	25.1	2.8	26.0	14.1	30.4	6/8	TP/M
Carbendazim	+	6.31	0.80	5.4	4.1	12.2	8/8	fungicide
Carbofuran-3-hydroxy	+	97.1	2.8	95.8	94.7	103.1	8/8	TP/M
Climbazole	+	157.4	3.9	140.9	61.6	409.6	8/8	fungicide
Cyprodinil	+	10.0	1.2	10.9	1.4	13.3	8/8	fungicide
DEET (Diethyltoluamide)	+	133.8	5.5	130.8	58.2	240.2	8/8	insect repellent
Difenoconazole	+	41.3	4.3	23.3	17.2	83.3	3/8	fungicide
Dimethachlor-ESA	+	1222	167	1154	945	1589	8/8	TP/M
Dimethoate	+	15.9	1.7	9.6	3.2	65.8	7/8	insecticide, acaricide
Fenamiphos	+	367.0	8.5	144.6	132.6	823.9	3/8	insecticide, nematicide
Fluconazole	+	156	11	156	135	170	8/8	fungicide
Fluometuron	+	38.6	2.3	17.3	12.7	132.2	8/8	herbicide
Metolachlor	+	4848	197	2353	2059	10,533	6/8	herbicide
Metolachlor-morpholinon	+	1986	129	1792	290	3304	4/8	TP/M
Penconazole	+	8.50	0.53	8.17	4.91	13.94	5/8	fungicide
Picaridin (Icaridin)	+	40.0	4.3	38.8	29.9	49.6	8/8	insect repellent
Prohexadione	+	59.2	3.8	46.0	42.2	92.0	7/8	plant growth regulator
Propiconazole	+	66.5	3.9	74.1	47.7	81.8	5/8	fungicide
Terbutryn	+	12.04	0.45	14.21	3.03	16.70	4/8	herbicide
Thiamethoxam	+	119	18	121	100	135	3/8	insecticide
Thiodicarb	+	8.45	0.20	7.06	6.78	11.53	3/8	insecticide
Tralkoxydim	+	4669	274	4709	4096	4906	8/8	herbicide
Fipronil	-	117	12	103	86	241	8/8	insecticide
Fludioxonil	-	29.0	4.0	29.8	9.6	59.6	8/8	fungicide
cis-1,2,3,6-Tetrahydrophthalimide	-	1597	106	1610	1398	1719	8/8	TP/M

Table S5. Wastewater concentrations (ng/L), standard deviations (SD) and frequency of detection (DF) of pesticides and their transformation products and/or metabolites (TP/M) in influent wastewater in **2017** of Athens, Greece.

Analyte	Polarity	Mean	SD	Median	Min.	Max.	DF	Pesticide class
Amitrole	+	535	60	571	411	610	7/7	herbicide
Anabasine	+	1930	186	1928	1389	2235	6/7	insecticide
Asulam	+	552	65	553	458	644	3/7	herbicide
Azoxystrobin	+	265	16	127	45	776	6/7	fungicide
Carbendazim	+	72.4					1/7	fungicide
Carbofuran-3-hydroxy	+	66.4	7.0	72.2	31.8	85.9	7/7	TP/M
Climbazole	+	277	14	226	197	415	7/7	fungicide
Cyprodinil	+	15.0	1.5	9.3	6.9	46.7	7/7	fungicide
DEET (Diethyltoluamide)	+	74.6	9.4	56.3	34.0	162.3	7/7	insect repellent
Difenoconazole	+	144.83	0.58	62.38	29.54	425.02	4/7	fungicide
Dimethachlor-ESA	+	520	26	547	136	746	7/7	TP/M
Fluometuron	+	127	13	115	96	187	5/7	herbicide
Metolachlor	+	63.2	9.2	46.4	28.4	145.7	7/7	herbicide
Phthalate-dimethyl	+	87	10	85	50	144	7/7	insect repellent
Picaridin (Icaridin)	+	24.9	3.0	25.6	17.7	34.2	7/7	insect repellent
Pirimiphos-methyl	+	7.18	0.49	5.67	3.66	13.72	4/7	insecticide, acaricide
Tebuconazole	+	44.8	1.7	44.8	43.7	46.0	2/7	fungicide
Terbutryn	+	5.45	0.45	5.45	5.13	5.77	2/7	herbicide
Thiodicarb	+	415	49	411	325	545	7/7	insecticide
Tralkoxydim	+	2689	286	2695	2078	3434	7/7	herbicide
cis-1,2,3,6-Tetrahydrophthalimide	-	1621	169	1841	618	1926	7/7	TP/M

Table S6. Wastewater concentrations (ng/L), standard deviations (SD) and frequency of detection (DF) of pesticides and their transformation products and/or metabolites (TP/M) in influent wastewater in **2018** of Athens, Greece.

Analyte	Polarity	Mean	SD	Median	Min.	Max.	DF	Pesticide class
Amitrole	+	864	102	873	733	971	6/7	herbicide
Azoxystrobin	+	1042	39	955	856	1269	5/7	fungicide
Azoxystrobin acid	+	367	21	240	142	975	5/7	TP/M
Carbendazim	+	2.30	0.16	8.38	7.05	14.40	7/7	fungicide
Carbofuran-3-hydroxy	+	202	18	195	189	237	6/7	TP/M
Climbazole	+	644	49	622	413	882	7/7	fungicide
Cyproconazole	+	204	25	165	115	428	5/7	fungicide
Cyprodinil	+	11.72	0.32	12.14	8.49	14.49	6/7	fungicide
DEET (Diethyltoluamide)	+	92.8	6.3	97.3	60.2	134.8	6/7	insect repellent
Difenoconazole	+	131	19	109	44	334	7/7	fungicide
Dimethachlor-ESA	+	937	81	963	759	1119	6/7	TP/M
Fluconazole	+	292	24	274	195	514	7/7	fungicide
Fluometuron	+	68.9	6.8	71.3	51.6	81.4	4/7	herbicide
Metolachlor	+	2655	387	2777	840	4225	4/7	herbicide
Metolachlor-morpholinon	+	32.19	0.75	29.09	18.99	51.59	4/7	TP/M
Napropamide	+	37.7	5.0	35.5	24.7	53.8	7/7	herbicide
Nicosulfuron	+	642	46	680	501	745	3/7	herbicide
Penconazole	+	85.0	6.9	98.3	17.1	148.9	6/7	fungicide
Phthalate-dimethyl	+	225	29	225	98	354	5/7	insect repellent
Prohexadione	+	271	40	290	175	368	5/7	plant growth regulator
Propamocarb	+	13.7	2.2	15.6	5.7	19.3	7/7	fungicide
Sethoxydim	+	1645	161	1634	1442	1877	6/7	herbicide
Thiamethoxam	+	210	14	189	76	401	6/7	insecticide
Tralkoxydim	+	2990	140	3079	2567	3305	6/7	herbicide
Fipronil	-	111	12	113	89	128	7/7	insecticide
Fludioxonil	-	6.55	0.89	6.27	5.35	8.15	5/7	fungicide
Dinoterb	-	143.3	3.3	122.4	41.1	211.0	7/7	herbicide
cis-1,2,3,6-Tetrahydrophthalimide	-	3344	238	3372	2917	3567	6/7	TP/M

Table S7. Wastewater concentrations (ng/L), standard deviations (SD) and frequency of detection (DF) of pesticides and their transformation products and/or metabolites (TP/M) in influent wastewater in **2019** of Athens, Greece.

Analyte	Polarity	Mean	SD	Median	Min.	Max.	DF	Pesticide class
Acetochlor	+	89.6	9.2	85.8	65.7	120.1	7/7	herbicide
Amitrole	+	193.6	3.2	176.9	112.5	266.0	7/7	herbicide
Azoxystrobin	+	16.5	1.4	9.0	3.7	35.9	6/7	fungicide
Carbofuran-3-hydroxy	+	69.5	9.7	70.3	39.4	89.0	7/7	TP/M
Carboxin	+	1407	168	1583	819	1820	3/7	fungicide
Climbazole	+	43.7	7.4	43.5	20.8	65.6	7/7	fungicide
DEET (Diethyltoluamide)	+	34.0	8.3	24.8	11.0	93.5	6/7	insect repellent
Difenoconazole	+	90.3	6.3	68.2	59.3	143.5	3/7	fungicide
Dimethachlor-ESA	+	239.0	9.5	248.3	111.3	415.0	7/7	TP/M
Fluconazole	+	33.0	3.2	33.3	26.6	39.0	7/7	fungicide
Fluometuron	+	5.22	0.23	5.19	3.20	8.69	5/7	herbicide
Metolachlor	+	32.7	1.4	26.1	8.4	74.5	5/7	herbicide
Penconazole	+	8.09					1/7	fungicide
Picaridin (Icaridin)	+	6.14	0.55	4.75	3.59	11.66	7/7	insect repellent
Propachlor-OXA	+	21.5	2.7	21.8	14.3	26.4	7/7	TP/M
Sethoxydim	+	377.3	55.3	348.8	272.5	485.4	7/7	herbicide
Tralkoxydim	+	670	38	644	380	919	7/7	herbicide
cis-1,2,3,6-Tetrahydrophthalimide	-	319	33	322	102	554	7/7	TP/M

Table S8. Wastewater concentrations (ng/L), standard deviations (SD) and frequency of detection (DF) of pesticides and their transformation products and/or metabolites (TP/M) in influent wastewater in **2020** of Athens, Greece.

Analyte	Polarity	Mean	SD	Median	Min.	Max.	DF	Pesticide class
Amitrole	+	168	15	131	85	414	7/7	herbicide
Asulam	+	11.6	1.2	12.0	6.5	14.1	5/7	herbicide
Atrazine-desisopropyl	+	19.4	2.0	20.8	13.9	23.8	7/7	TP/M
Azoxystrobin	+	107.2	4.4	69.2	12.2	258.0	6/7	fungicide
Carbendazim	+	6.60	0.48	2.91	2.48	18.09	4/7	fungicide
Carbofuran-3-hydroxy	+	75	11	75	48	116	7/7	TP/M
Climbazole	+	37.2	2.7	36.3	16.8	55.8	7/7	fungicide
DEET (Diethyltoluamide)	+	17.9	1.7	17.7	11.1	29.1	7/7	insect repellent
Difenoconazole	+	64.3	3.9	14.6	11.9	17.4	2/7	fungicide
Dimethachlor-ESA	+	100	14	90	37	184	7/7	TP/M
Fluconazole	+	24.8	1.0	24.8	18.0	36.5	7/7	fungicide
Fluometuron	+	313	34	368	154	416	3/7	herbicide
Metolachlor	+	32.4	4.4	17.6	9.1	94.8	5/7	herbicide
Picaridin (Icaridin)	+	9.2	1.0	9.8	3.6	12.5	7/7	insect repellent
Pirimiphos-methyl	+	4.64	0.38	2.94	1.41	11.91	6/7	insecticide, acaricide
Prohexadione	+	14.7	1.4	14.5	11.6	21.7	7/7	plant growth regulator
Propachlor-OXA	+	3.0	3.0	242.0	132.2	415.5	6/7	TP/M
Propamocarb	+	1.71	0.22	1.59	0.96	3.06	7/7	fungicide
Sethoxydim	+	488	14	475	340	695	6/7	herbicide
Terbutryn	+	7.5					1/7	herbicide
Thiamethoxam	+	29.6	2.5	20.6	12.8	64.6	4/7	insecticide
Tralkoxydim	+	186	13	186	51	284	7/7	herbicide
Fipronil	-	38.2	4.9	38.2	34.7	41.7	2/7	insecticide
Fludioxonil	-	6.97	0.67	3.40	2.45	15.06	3/7	fungicide
cis-1,2,3,6-Tetrahydrophthalimide	-	1512	167	1498	1179	1812	6/7	TP/M

Table S9. Pesticides determined from 2014 to 2020 in influent wastewater samples of Athens, Greece. Uses and regulatory status (https://ec.europa.eu/food/plant/pesticides/eu-pesticides-database/active-substances/, https://pesticidecompendium.bcpc.org, https://echa.europa.eu/, http://sitem.herts.ac.uk/aeru/iupac/index.htm).

Compound	Plant protection active substance<br>Current approval status under Reg. (EC) No 1107/2009 (EC legislation)	Biocidal active substance<br>Current approval status under Regulation (EU) 528/2009	Other uses/<br>Other legislation
Acetochlor	Not approved (2008/934, Reg. (EU) No 1372/2011)	-	-
Ametryn	Not approved (2002/2076)	-	Registered under the REACH Regulation.<br>Used in formulation or re-packing and in manufacturing.<br>Release to the environment of this substance can occur from industrial use: formulation of mixtures.
Amitrole	Not approved (01/21/EC, 2010/77/EU, Reg (EU) 2015/408, Reg. (EU) 2015/1885, Reg. (EU) 2016/871, Reg. (EU) No 540/2011)	-	Registered under the REACH Regulation.<br>Used in the following activities or processes at workplace: laboratory work.<br>Other release to the environment of this substance is likely to occur from: indoor use (e.g., machine wash liquids/detergents, automotive care products, paints and coating or adhesives, fragrances and air fresheners).
Anabasine	-	-	Included in the pesticide database (https://pesticidecompendium.bcpc.org).<br>Occurrence related to tobacco.
Asulam sodium	Pending<br>Originally not approved:<br>Reg (EC) 1045/2011 (2008/934)	-	-
Atrazine & TP/M (atrazine-desethyl and atrazine-desisopropyl)	Not approved (2004/248/EC)	-	Registered under the REACH Regulation.<br>Used at industrial sites.
Azoxystrobin & TP/M (azoxystrobin acid)	Approved (1998/47/EC, 2007/21/EC, 2010/55/EU, Reg. (EU) 2018/155, Reg. (EU) No 2019/291, Reg. (EU) No 540/2011, Reg. (EU) No 703/2011)	-	-
Captan & TP/M (*cis*-1,2,3,6-	Approved	-	Registered under the REACH Regulation.

Tetrahydrophthalimide)	(07/5/EC, Reg. (EU) 2018/917, Reg. (EU) 2019/707, Reg. (EU) 2020/869, Reg. (EU) No 540/2011)		Used in articles, by professional workers (widespread uses), in formulation or re-packing and at industrial sites.
Carbendazim	Not approved (2006/135/EC, 2010/70/EC, 2011/58/EU, Reg (EU) 2015/408, Reg. (EU) No 540/2011, Reg. (EU) No 542/2011)	Approved as preservative. Used for films preservation, preservation for construction materials.	Registered under the REACH Regulation.<br>Used in formulation or re-packing.
Carbofuran & TP/M (carbofuran-3-hydroxy)	Not approved (2007/416)	-	-
Carboxin	Approved (2011/52/EU, Reg. (EU) No 2018/1266, Reg. (EU) No 2019/324, Reg. (EU) No 540/2011)	-	-
Climbazole	Not included in the EU pesticides database	-	Registered under the REACH Regulation.<br>Used in the following products: cosmetics and personal care products.<br>Other release to the environment of this substance is likely to occur from indoor use as processing aid.<br>Included in the pesticide database (https://pesticidecompendium.bcpc.org/)
Cyproconazole	Approved (2011/56/EU, Reg. (EU) No 540/2011)	Approved as wood preservative (approval currently expired).	Registered under the REACH Regulation.
Cyprodinil	Approved (06/64/EC, Reg. (EU) 2018/524, Reg. (EU) 2019/168, Reg. (EU) 2020/421, Reg. (EU) 2021/566, Reg. (EU) No 540/2011, Reg. (EU) No 678/2014)	-	-
DEET	Not included in the EU pesticides database	Approved for pest control (Repellents and attractants).	-
Difenoconazole	Approved (2008/69, Reg. (EU) 2018/1796, Reg. (EU) 2019/1589, Reg. (EU) 2020/1511, Reg. (EU) No 1100/2011, Reg. (EU) No 540/2011)	-	-
Dimethachlor & TP/M (dimethachlor-ESA)	Approved (2009/77/EC, Reg. (EU) No 540/2011)	-	-

Dimethoate	Not approved (07/25/EC, Reg. (EU) 2018/917, Reg. (EU) 2019/1090, Reg. (EU) 2019/707, Reg. (EU) No 540/2011)	-	-
Dinoterb	Not approved (98/269/EC)	-	-
Fenamiphos	Not approved (06/85/EC, Reg. (EU) 2015/415, Reg. (EU) 2018/917, Reg. (EU) 2019/707, Reg. (EU) 2020/1246, Reg. (EU) 2020/869, Reg. (EU) No 540/2011)	-	-
Fipronil	Not approved (07/52/EC, 2010/21/EU, Reg (EU) 2015/408, Reg. (EU) 2016/2035, Reg. (EU) No 540/2011, Reg. (EU) No 781/2013)	Approved as insecticides, acaricides and products to control other arthropods.	Registered under the REACH Regulation.
Fluconazole	Not included in the EU pesticides database	-	Used as antifungal medicine. Listed in the NORMAN Suspect List Exchange (https://pubchem.ncbi.nlm.nih.gov/)
Fludioxonil	Approved (2007/76, Reg. (EU) 2018/1262, Reg. (EU) 2019/1589, Reg. (EU) 2020/1511, Reg. (EU) No 540/2011)	Approved as preservative. Used as<br>- Film preservative,<br>- Fibre, leather, rubber and polymerised materials preservatives,<br>- Construction material preservatives.	-
Fluometuron	Approved (2011/57/EU, Reg. (EU) 2020/2007, Reg. (EU) No 2018/1266, Reg. (EU) No 540/2011)	-	Registered under the REACH Regulation.<br>Used in articles, in formulation or re-packing and at industrial sites.<br>Other release to the environment of this substance is likely to occur from outdoor use in long-life materials with low release rate (e.g., metal, wooden and plastic construction and building materials) and indoor use in long-life materials with low release rate (e.g., flooring, furniture, toys, construction materials, curtains, footwear, leather products, paper and cardboard products, electronic equipment).<br>This substance can be found in complex articles, with no release intended: vehicles and machinery, mechanical appliances and electrical/electronic products (e.g.,

			computers, cameras, lamps, refrigerators, washing machines).
Flutolanil	Approved (2008/108, Reg. (EU) 2019/168, Reg. (EU) 2019/2094, Reg. (EU) 2021/52, Reg. (EU) No 540/2011)	-	-
Irgarol	Not included in the EU pesticides database	-	Used in the past as booster biocide agent, added in copper-based antifouling paints, applied in surfaces submerged in water to prevent biofouling.
Metalaxyl	Approved (2010/28/EU, Reg. (EU) 2015/1885, Reg. (EU) 2017/2069, Reg. (EU) No 540/2011)	-	-
Metolachlor & TP/M (metolachlor-morpholinon)	Approved (S-Metolachlor) (Reg EU 2021/745, 05/3/EC, Reg. (EU) No 540/2011)	-	-
Napropamide	Approved (2010/83/EU, Reg. (EU) 2018/670, Reg. (EU) No 540/2011)	-	-
Nicosulfuron	Approved (2008/40, Reg. (EU) 2018/1796, Reg. (EU) 2019/1589, Reg. (EU) 2020/1511, Reg. (EU) No 540/2011)	-	-
Penconazole	Approved (2009/77/EC, 2010/34/EU, Reg. (EU) No 540/2011)	-	-
Phthalate dimethyl	Not included in the EU pesticides database	-	Registered under the REACH Regulation. Used in the following products: perfumes and fragrances, cosmetics and personal care products, coating products, air care products and washing & cleaning products. Other release to the environment of this substance is likely to occur from indoor use (e.g., machine wash liquids/detergents, automotive care products, paints and coating or adhesives, fragrances and air fresheners), outdoor use and indoor use in close systems with minimal release (e.g., cooling liquids in refrigerators, oil-based electric heaters).
Picaridin (Icaridin)	Not included in the EU pesticides database	Approved as repellent and attractant.	Registered under the REACH Regulation.

Pirimiphos methyl	Approved (07/52/EC, Reg. (EU) 2018/917, Reg. (EU) 2019/707, Reg. (EU) 2020/869, Reg. (EU) No 540/2011)	-	-
Prohexadione	Approved (2000/50/EC, 2007/21/EC, 2010/56/EU, Reg. (EU) No 2019/291, Reg. (EU) No 540/2011, Reg. (EU) No 702/2011)	-	-
Propachlor & TP/M (propachlor-OXA)	Not approved (2008/742)	-	Included in the pesticide database (http://sitem.herts.ac.uk/aeru/iupac/index.htm). A pre-emergence herbicide for control of annual grasses and some broad-leaved weeds.
Propamocarb	Approved (7/25/EC, Reg. (EU) 2018/917, Reg. (EU) 2019/707, Reg. (EU) 2020/869, Reg. (EU) No 540/2011)	-	-
Propiconazole	Not approved (03/70/EC, Reg. (EU) 2016/2016, Reg. (EU) 2018/1865, Reg. (EU) No 540/2011, Reg. (EU) No 823/2012, Reg. (EU) 2018/84)	Approved as preservative. Used for preservation films, wood preservation, preservation of fibres, leather, rubber, or polymers.	Registered under the REACH Regulation. Used in formulation or re-packing.
Sethoxydim	Not approved	-	Included in the pesticide database (http://sitem.herts.ac.uk/aeru/iupac/index.htm). A post-emergence, selective, annual and perennial grass weed herbicide.
Tebuconazole	Approved (2008/125, Reg. (EU) 2019/707, Reg. (EU) 2020/1160, Reg. (EU) No 540/2011, Reg. (EU) No 921/2014)	Approved as preservative. Used as - Film preservative, - Fibre, leather, rubber and polymerised materials preservatives, - Construction material preservatives.	Registered under the REACH Regulation.
Terbacil	Not approved (2002/2076)	-	Included in the pesticide database (http://sitem.herts.ac.uk/aeru/iupac/index.htm). Herbicide for control of annual grasses, broad-leaved weeds and some perennial weeds that is used on a range of crops including top fruit, lucerne and some herbs.

Terbutryn	Not approved (2002/2076)	Under evaluation as preservative for uses as:<br>- Film preservative,<br>- Fibre, leather, rubber and polymerised materials preservatives,<br>- Construction material preservatives.	-
Thiamethoxam	Not approved (07/6/EC, 2010/21/EU, Reg. (EU) No 2018/524, Reg. (EU) No 2018/785, Reg. (EU) No 485/2013, Reg. (EU) No 487/2014, Reg. (EU) No 540/2011)	Approved as wood preservative in the past (expired approval). Currently approved as insecticides, acaricides and products to control other arthropods.	Registered under the REACH Regulation.<br>Used in formulation or re-packing and in manufacturing.
Thiodicarb	Not approved	-	-
Tralkoxydim	Not approved (2008/107, Reg. (EU) No 540/2011)	-	-
